# Thiostrepton, a resurging drug inhibiting the stringent response to counteract antibiotic-resistance and expression of virulence determinants in *Neisseria gonorrhoeae*

**DOI:** 10.3389/fmicb.2023.1104454

**Published:** 2023-02-23

**Authors:** Adelfia Talà, Matteo Calcagnile, Silvia Caterina Resta, Antonio Pennetta, Giuseppe Egidio De Benedetto, Pietro Alifano

**Affiliations:** ^1^Department of Biological and Environmental Sciences and Technologies, University of Salento, Lecce, Italy; ^2^Laboratory of Analytical and Isotopic Mass Spectrometry, Department of Cultural Heritage, University of Salento, Lecce, Italy

**Keywords:** antibiotic-resistance, thiopeptide antibiotics, gonococcus, stringent response, transcriptome analysis

## Abstract

Due to the increased resistance to all available antibiotics and the lack of vaccines, *Neisseria gonorrhoeae* (the gonococcus) poses an urgent threat. Although the mechanisms of virulence and antibiotic resistance have been largely investigated in this bacterium, very few studies have addressed the stringent response (SR) that in pathogenic bacteria controls the expression of genes involved in host-pathogen interaction and tolerance and persistence toward antibiotics. In this study, the results of the transcriptome analysis of a clinical isolate of *N. gonorrhoeae*, after induction of the SR by serine hydroxamate, provided us with an accurate list of genes that are transcriptionally modulated during the SR. The list includes genes associated with metabolism, cellular machine functions, host-pathogen interaction, genome plasticity, and antibiotic tolerance and persistence. Moreover, we found that the artificial induction of the SR in *N. gonorrhoeae* by serine hydroxamate is prevented by thiostrepton, a thiopeptide antibiotic that is known to interact with ribosomal protein L11, thereby inhibiting functions of EF-Tu and EF-G, and binding of pppGpp synthase I (RelA) to ribosome upon entry of uncharged tRNA. We found that *N. gonorrhoeae* is highly sensitive to thiostrepton under *in vitro* conditions, and that thiostrepton, in contrast to other antibiotics, does not induce tolerance or persistence. Finally, we observed that thiostrepton attenuated the expression of key genes involved in the host-pathogen interaction. These properties make thiostrepton a good drug candidate for dampening bacterial virulence and preventing antibiotic tolerance and persistence. The ongoing challenge is to increase the bioavailability of thiostrepton through the use of chemistry and nanotechnology.

## Introduction

Various terms, with some important differences, have been proposed to describe bacterial non-mutational and non-inheritable resistance to antibiotic treatments, such as bacterial tolerance, bacterial persistence, phenotypic resistance, phenotypic heterogeneity, adaptive resistance, collective tolerance, collective persistence, recalcitrance, or, collectively, resilience (Brauner et al., 2016; Carvalho et al., 2019; Dewachter et al., 2019). This phenomenon, which is often responsible for failure of antibiotic therapy, involves a plurality of mechanisms and has two general characteristics: it is transient and occurs in response to certain environmental or metabolic conditions (Michiels et al., 2016; Andersson et al., 2019; Kaldalu et al., 2020).

On a mechanistic point of view, it is crucial to distinguish between tolerance and persistence (Brauner et al., 2016). While the term “resistance” is clearly defined as the inherited ability of bacteria to grow at high concentrations of an antibiotic, regardless of the duration of treatment, and is quantified by the minimum inhibitory concentration (MIC), the distinction between the terms “tolerance” and “persistence” is sometimes ambiguous (Brauner et al., 2016; Balaban et al., 2019). Here we refer to tolerance as the slower killing (reduced killing rate) of the bacterial population as a whole after exposure to bactericidal antibiotic without a change in the MIC, and to persistence as a phenomenon mediated by a small fraction of the population that is slowly killed resulting in a biphasic killing curve: after an initial rapid decline in bacterial counts, a much slower decline is observed, due to poor killing of “persister” cells (Brauner et al., 2016). Therefore, persistence could be considered a form of tolerance induced in a fraction of the population, and implies the existence of phenotypic heterogeneity in the population.

Tolerance and resistance are often achieved by slowing down an essential bacterial process. Antibiotics that target active growth processes such as cell wall assembly, transcription, protein synthesis or DNA replication kill tolerant or persistent bacteria less efficiently. For example, tolerance to β-lactam antibiotics is induced by slowing down cell wall assembly and is proportional to the rate of bacterial growth (Tuomanen et al., 1986). Tolerance and resistance can be considered two modes of the same “persistent phenotype,” which is considered to be the main cause of the persistent and relapsing course of many bacterial infections (Andersson et al., 2019; Pacios et al., 2020).

Under *in vivo* conditions, various environmental and metabolic conditions trigger stress signals that induce the persistent phenotype including the transition to the stationary phase, exposure to sub-lethal antibiotic concentrations, the internalization of bacteria by host immune and non-immune cells, and growth in the biofilm (Harms et al., 2016). Thus, the persistent phenotype is mostly controlled by general stress signaling pathways (Kaldalu et al., 2020), and a plethora of mechanisms have been proposed to be involved in tolerance and persistence including toxin-antitoxin systems, the stringent response (SR), the quorum sensing (QS), drug efflux pumps, the SOS response, the oxidative stress response, and the stationary phase RpoS sigma factor regulon (Trastoy et al., 2018; Kaldalu et al., 2020). In particular, the SR, which is triggered by the stress alarmone guanosine pentaphosphate/tetraphosphate [(p)ppGpp], appears to play a central role in the induction of tolerance and persistence (Fernández et al., 2011; Pacios et al., 2020).

The biosynthesis of (p)ppGpp is catalyzed by proteins belonging to the RelA/SpoT homolog (RSH) superfamily, and is triggered not only by amino acid limitation as initially identified in *Escherichia coli*, but also in response to a wide range of signals including impairment of fatty acid metabolism, cell wall stress, alkaline shock, osmotic shock and temperature shift (Potrykus and Cashel, 2008; Irving and Corrigan, 2018; Ronneau and Hallez, 2019). The SR is not only involved in metabolic or environmental stresses responses by reprogramming gene expression (Potrykus and Cashel, 2008; Dalebroux and Swanson, 2012), but it is also used by pathogenic bacteria to regulate the expression of genes involved in host-pathogen interaction, and antibiotic tolerance and persistence (Dalebroux et al., 2010; Pacios et al., 2020). Therefore, the SR represents a good target for new drugs.

To understand the basis of inhibition of (p)ppGpp synthesis, it is important to understand the activity, distribution and phylogenesis of the RSH superfamily proteins (Potrykus and Cashel, 2008; Dalebroux et al., 2010; Atkinson et al., 2011; Dalebroux and Swanson, 2012; Irving and Corrigan, 2018; Ronneau and Hallez, 2019; Jimmy et al., 2020). RSH proteins can be divided into three groups: (i) long RSHs, which include Rel, RelA and SpoT containing (p)ppGpp synthetase (Syn), hydrolase domain (HD), TGS and ACT domains; (ii) small alarmone synthetases (SASs) containing only Syn domain; and (iii) Small alarmone hydrolases (SAHs) containing only HD. Two long RSHs, RelA and SpoT, are found in γ-and β-proteobacteria (Potrykus and Cashel, 2008; Dalebroux et al., 2010; Atkinson et al., 2011; Dalebroux and Swanson, 2012; Irving and Corrigan, 2018; Ronneau and Hallez, 2019; Jimmy et al., 2020). In *E. coli*, RelA, a ribosome-associated proteins, senses deacylated tRNA bound in the ribosomal A-site under conditions of amino acid limitation, and uses ATP and GTP (or GDP) to synthesize the alarmone pppGpp (or ppGpp) by the Syn domain. Then, pppGpp is rapidly converted into ppGpp by the pppGpp phosphohydrolase (GPP). SpoT, a second long RHS, which is not associated with the ribosome and is bifunctional with weak ppGpp synthetase activity and strong ppGpp degrading activity, is responsible for ppGpp hydrolysis by the HD. The HD is inactive in *E. coli* RelA (Potrykus and Cashel, 2008; Dalebroux et al., 2010; Atkinson et al., 2011; Dalebroux and Swanson, 2012; Irving and Corrigan, 2018; Ronneau and Hallez, 2019; Jimmy et al., 2020).

Among antibiotics that can inhibit induction of the SR, some thiopeptides that interfere with translation, such as thiostrepton, nosiheptide and micrococcin, are good candidates (Kudrin et al., 2017, 2018; Polikanov et al., 2018; Bailly, 2022). The thiopeptide binding site on the ribosomal large subunit sterically overlaps with the binding site of translation factors, such as initiation factor 2 (IF2), elongation factor Tu (EF-Tu) and elongation factor G (EF-G) (Walsh et al., 2010; Polikanov et al., 2018). As a consequence, thiopeptides inhibit IF-2-dependent initiation complex formation, EF-Tu-dependent delivery of the amino acyl-tRNA to the A-site, and accommodation of EF-G, leading to inhibition of the translocation step of translation (Just-Baringo et al., 2014; Polikanov et al., 2018).

Interestingly, thiostrepton binds the ribosomal large subunit in a cleft formed between the N-terminal domain of ribosomal protein L11 and helices H43 and H44 of the 23S rRNA (Harms et al., 2008). By interacting with L11 protein, which mediates pppGpp synthase I (RelA) binding to ribosome upon entry of uncharged tRNA at the A site, and inhibiting functions of EF-Tu and EF-G, thiostrepton reduces the RelA-dependent synthesis of pppGpp in both Gram-positive and Gram-negative bacteria (Cundliffe and Thompson, 1981; Fehr and Richter, 1981). Using a biochemical system from purified *E. coli*, Kudrin and coworkers showed that thiostrepton and nosiheptide inhibit RelA activation by the A-site tRNA (Kudrin et al., 2017, 2018).

These premises led us to study in more detail the ability of thiostrepton, an antibiotic that was discovered in 1955 (Donovick et al., 1955) and is mostly used topically in veterinary medicine due to its low solubility, to interfere with the induction of the SR, tolerance and persistence in *Neisseria gonorrhoeae* (the gonococcus). *N. gonorrhoeae* is an obligate human pathogen and the etiological agent of gonorrhea, which each year causes an estimated 10^6^ new cases worldwide (Virji, 2009). This microorganism was recently classified by the World Health Organization as an urgent threat, due to both the rise of resistance to all available antibiotics and the lack of any viable vaccine candidates (Unemo et al., 2019).

In this study we show that *N*. *gonorrhoeae*, despite being Gram-negative like *E. coli* which is poorly sensitive to thiostrepton (Kelly et al., 1959a,b; Bailly, 2022), is extremely sensitive to thiostrepton, and that treatment with this antibiotic does not induce tolerance or persistence. We used RNA-seq to characterize the whole transcript profile of gonococci: (i) following induction of the SR with serine hydroxamate, which is a competitive inhibitor of seryl-tRNA synthetase (Tosa and Pizer, 1971a), (ii) following treatment with thiostrepton, or (iii) Combined treatment with serine hydroxamate and thiostrepton. Overall, the data provided insight into the regulation of the expression of genes associated with metabolism, cellular machine functions, virulence, genomic plasticity and adaptive resistance, including toxin-antitoxin modules, during the SR and/or thiostrepton treatment in this important pathogen.

## Results

### Sensitivity of gonococci to thiostrepton, killing curves, and adaptive resistance after exposure to antibiotics

**Figure 1:**
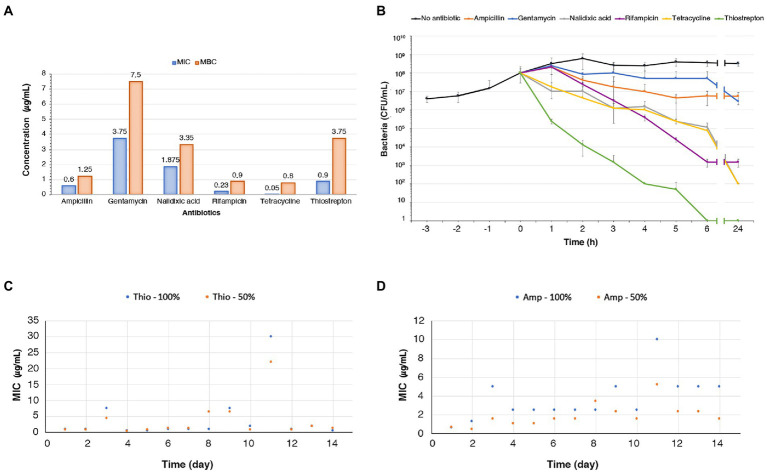
MIC, MBC, antibiotic killing assays, and adaptive resistance experiments. **(A)** Histogram of MICs and MBCs of thiostrepton, ampicillin, gentamicin, nalidixic acid, rifampicin, tetracycline against *N. gonorrhoeae* T9 grown in GC broth. The MIC/MBC procedures were repeated three times for each antibiotic on independent assays. **(B)** Time-kill curve experiments with thiostrepton (3.75 μg/mL), ampicillin (1.25 μg/mL), gentamicin (7.5 μg/mL), nalidixic acid (3.35 μg/mL), rifampicin (0.9 μg/mL), tetracycline (0.8 μg/mL), added to the bacterial suspension at a concentration corresponding to their MBC value (numbers in brackets). The mean values of three independent experiments with standard deviation are shown. **(C,D)** *N. gonorrhoeae* T9 was inoculated sequentially into 14 sets of tubes containing GC broth with increasing concentrations (50% or 100% as indicated) of thiostrepton **(C)** or ampicillin **(D)**. Bacteria growing at the highest antibiotic concentration in each set of tubes were used to inoculate the following set with the same antibiotic for a total of 14 passages. After each passage, the MIC of thiostrepton **(C)** or ampicillin **(D)** was determined.

In this study we started with the evaluation of the sensitivity of *N. gonorrhoeae* to thiostrepton since, as far as we know, the only finding in the literature on thiostrepton sensitivity of gonococci dates back to 1967 (Korzybski et al., 1967). After determining growth curve of *N. gonorrhoeae* strain T9 (Martin et al., 1986) in gonococcal (GC) broth supplemented with Polyvitox, its sensitivity to ampicillin, gentamicin, nalidixic acid, rifampicin, tetracycline, and thiostrepton was evaluated. The strain showed a high sensitivity to all antibiotics. Minimum inhibitory concentration (MIC) and minimum bactericidal concentration (MBC) values are shown in Figure 1A. The MIC value of less than 1 μg/mL (0.54 μM) for thiostrepton, a complex cyclic thiopeptide that is poorly effective against most Gram-negative bacteria, is remarkable and consistent with previous results (Korzybski et al., 1967). The same MIC value was also found with *N. gonorrhoeae* strain T2 (Martin et al., 1986). Specifically, two different thiostrepton stock solutions, one dissolved in dimethyl sulphoxide and the other in Pluronic F-127, were used to determine the MIC/MBC values in T2 and T9 gonococcal strains. No significant differences were observed in MIC and MBC values in each distinct experimental condition, and between the two gonococcal strains.

After determining sensitivity of *N. gonorrhoeae* strain T9 to thiostrepton and other antibiotics, antibiotic killing assays were carried out (Figure 1B). Bacteria were grown to mid log phase up to a concentration of about 10^8^ CFU/mL, and then ampicillin, gentamicin, nalidixic acid, rifampicin, tetracycline, or thiostrepton were added at a final concentration corresponding to their respective MBC values. CFU were determined at different time points and compared to CFU of untreated bacteria. The six antibiotics exhibited bactericidal effects with some differences.

When treated with ampicillin, the bacteria continued to grow during the first hour, albeit to a lesser extent, and then a weak bactericidal effect was observed over time up to 5 h, with a reduction in the number of CFU to about 5 × 10^6^ (Figure 1B). No further reduction was observed over the next hours, but rather a weak increase in the number of CFU. The decline followed by the leveling of the number of CFU after treatment with ampicillin was indicative of persistence of gonococcal strain T9. This finding is consistently with old results showing that a small but significant fraction of bacteria in a gonococcal population responded in a bacteriostatic rather than a bactericidal way upon ampicillin treatment, despite rather low MIC values (Elmros et al., 1979).

Gentamicin started to kill bacteria at a very slow rate after 1 h of incubation reducing the CFU number to approximately 6 × 10^7^ after 6 h of incubation (Figure 1B). After this time, the decline in the number of CFU was more pronounced. This result, which was indicative of tolerance of strain T9 against gentamicin, was rather surprising because gentamicin is considered bactericidal and usually yields substantial and rapid killing against aerobic bacteria (Young and Hewitt, 1973), and is recommended for the treatment of gonorrhea as a second-line agent (Burmeister et al., 2020). With rifampicin the killing started after 1 h, and the killing rate was steady and moderate up to 6 h. No further reduction was observed from 6 h to 24 h of incubation. Similar to ampicillin, the rifampicin kill curve profile is indicative of persistence of gonococcal strain T9. Finally, gonococci treated with nalidixic acid and tetracycline showed similar killing curves, with about 10^2^ CFU after 24 h of incubation.

With thiostrepton, killing began almost immediately after adding the antibiotic, and the killing rate was higher than that seen with the other antibiotics, and no surviving bacteria were detected after 6 h. The evidence that thiostrepton was able to kill gonococci without residual survivors in time-kill curves supported the hypothesis that it was unable to induce tolerance or persistence phenomena, unlike β-lactams and other classes of antibiotics.

Adaptive antibiotic resistance is a transient phenomenon that can emerge when populations of bacteria are exposed to gradual increases in subinhibitory antibiotic concentrations. The hallmarks of this phenomenon are the rapid emergence of the resistant phenotype and rapid returning to the non-resistant phenotype when the antibiotic is removed from the medium. Induction of adaptive antibiotic resistance involves epigenetic mechanisms and heterogeneity of gene expression pattern, mostly with changes in porin and efflux pump expression (Adam et al., 2008; Motta et al., 2015). This phenomenon should be distinguished from heritable genetic resistance evolved under the pressure of subinhibitory antibiotic concentrations (Toprak et al., 2011), in which bacteria do not revert to the non-resistant phenotype after antibiotic removal. We sought to understand whether thiostrepton was able to induce adaptive resistance. To this purpose, we exposed *N. gonorrhoeae* strains T2 and T9 to successive steps of increasing concentration of thiostrepton. Ampicillin was used as a control.

This experiment was performed using a gradient approach (Jahn et al., 2017). Gonococci were used to inoculate sequentially 14 sets of tubes containing GC broth with increasing concentrations (50% or 100%) of each antibiotic. Bacteria growing at the highest antibiotic concentration in each set of tubes were used to inoculate the following set with the same antibiotic for a total of 14 passages. After each passage, the MIC of each antibiotic was determined.

The results obtained with the T9 strain demonstrated that the thiostrepton MIC values remained almost similar to the initial strain values in most passages (days 1, 2, 4, 5, 6, 7, 10, 12, 13, and 14), with some transient increases observed on days 3, 9 and 11, likely due to selection of resistant mutants with reduced fitness in subsequent steps (Figure 1C). An increase was also observed on day 8 only in the tube set with 50% increase in antibiotic concentration. At the end of 14 passages, thiostrepton MIC was similar to baseline, supporting the absence of thiostrepton-induced adaptive resistance phenomena. Similar results were obtained with the T2 strain in the tube set with 50% increase in antibiotic concentration (Supplementary Figure S1). However, in the tube set with 100% increase in antibiotic concentration, a clear increase in thiostreptone resistance was observed on day 8, but the bacteria were unable to grow after this passage, possibly due to selection of a deleterious mutation conferring genetic resistance to thiostrepton. Conversely, when the T9 strain was cultured in the presence of ampicillin, we recorded a slight and progressive increase in ampicillin resistance, more pronounced in the tube set with 100% increase in antibiotic concentration than in the tube set with 50% increase (Figure 1D). At the end of 14 passages, ampicillin MIC values were increased 8.33-fold in the tube set with 100% increase in antibiotic concentration, and 2.26-fold in the tube set with 50% increase compared to the initial MIC values. This result was compatible with either ampicillin-induced non-mutational adaptive resistance or mutational adaptive evolution.

### Sensitivity of gonococci to serine hydroxamate and induction of the stringent response

Since there is evidence that tolerance and persistence to antibiotic treatment may be linked to the induction of the SR (Fernández et al., 2011; Pacios et al., 2020), and since thiostrepton interferes with its trigger (Cundliffe and Thompson, 1981; Fehr and Richter, 1981; Kudrin et al., 2017), the next step was to analyze the SR that in the gonococcus was studied in the context of bacterial stress (Fisher et al., 2005; Wu et al., 2010; Zielke et al., 2015; Bauer et al., 2017; Churchward et al., 2018; Quillin et al., 2018; Welker et al., 2021).

To trigger the SR, we used bacteriostatic serine hydroxamate, which is a competitive inhibitor of seryl-tRNA synthetase (Tosa and Pizer, 1971a). The transcriptome of *N. gonorroheae*, after induction of the SR, was then analyzed by RNA-seq experiments.

**Figure 2:**
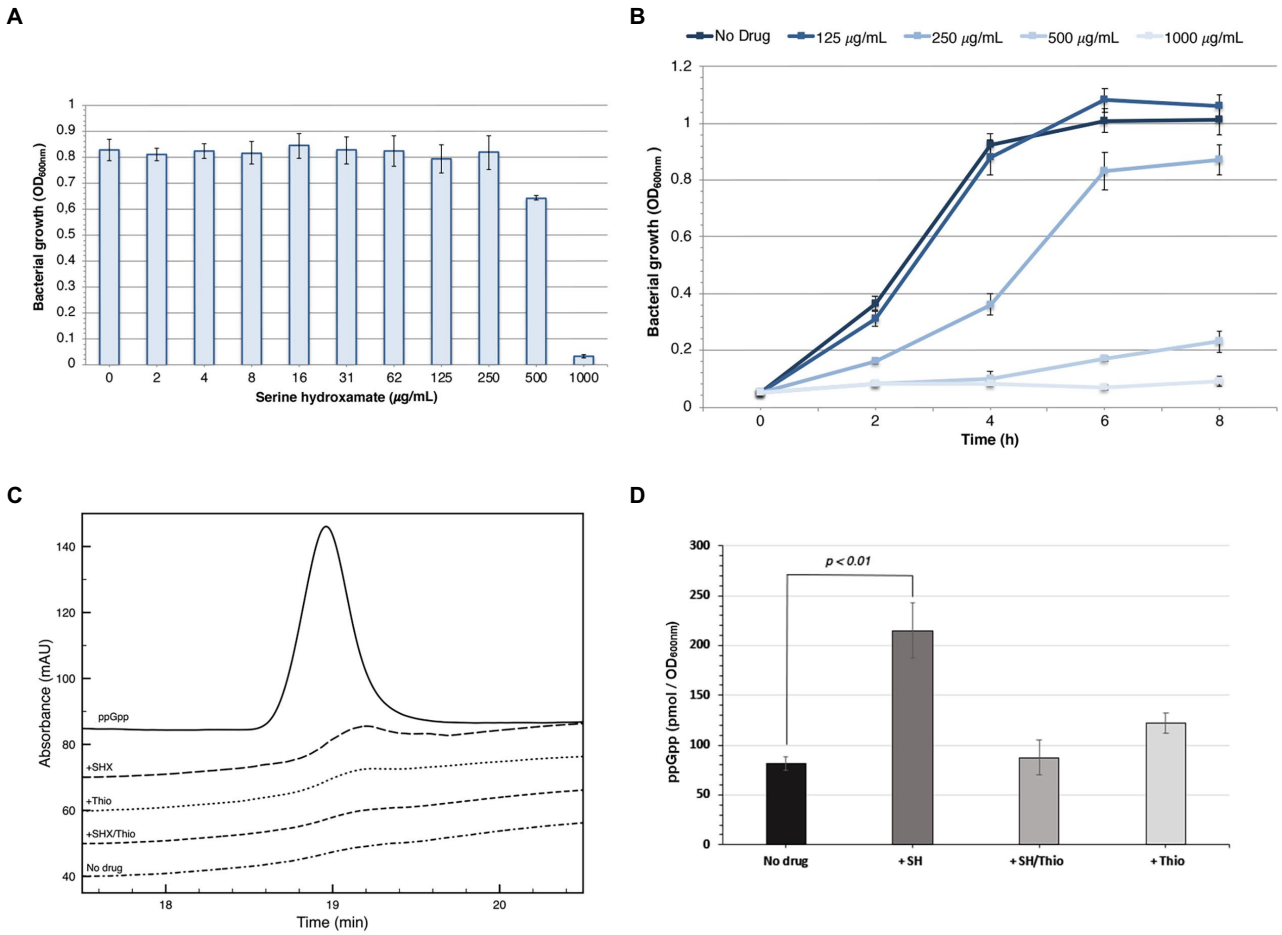
Sensitivity to serine hydroxamate and ppGpp levels. **(A)** MIC of serine hydroxamate against *N. gonorrhoeae* T9 grown in GC broth. The MIC value is 1 mg/mL. The results are the average of three separate experiments and standard deviation is shown. **(B)** Growth curve of *N. gonorrhoeae* T9 in the presence of different concentrations of serine hydroxamate. The mean values of three independent assays with standard deviation are shown. **(C,D)** Intracellular levels of ppGpp in *N. gonorrhoeae* T9 cells after serine hydroxamate or serine hydroxamate/thiostrepton treatments. Bacteria were grown to logarithmic phase (OD_600nm_ = 0.5) in GC broth and then treated with serine hydroxamate (1 mg/mL) and/or thiostrepton (3.75 μg/mL) for 30 min. Representative HPLC chromatograms **(C)** and ppGpp levels **(D)** are shown. No drug: control untreated bacteria; + SH: bacteria treated with serine hydroxamate; +Thio: bacteria treated with thiostrepton; + SH/Thio: bacteria treated with serine hydroxamate and thiostrepton. The mean values of three independent assays with standard deviation are shown in panel F. *p* values were determined by PAST software.

Preliminarily, we determined the sensitivity of *N. gonorrhoeae* T9 to serine hydroxamate in GC broth with Polyvitox. MIC experiments showed that bacterial growth was moderately inhibited at a serine hydroxamate concentration of 500 μg/mL (4.16 mM), and completely inhibited at 1 mg/mL (8.32 mM; Figure 2A). Figure 2B shows the growth curve in the presence of different concentrations of the drug.

To assess whether treatment with serine hydroxamate actually induced the accumulation of (p)ppGpp in *N. gonorrhoeae* T9, a direct measurement of ppGpp levels by anion exchange chromatography with UV detection was performed. Bacteria were grown to logarithmic phase (OD_600nm_ = 0.5) in GC broth and then treated with serine hydroxamate (at the concentration of 1 mg/mL, corresponding to the MIC value) and/or thiostrepton (at the concentration of 3.75 μg/mL, corresponding to the MBC value) for 30 min. Serine hydroxamate treatment of bacterial cultures resulted in clearly induced accumulation of ppGpp (Figures 2C,D). The basal ppGpp level (~81.6 pmol/OD_600 nm_) significantly increased (*p* value = 0.0094) about 2.6-fold (~215 pmol/OD_600 nm_) after serine hydroxamate exposure. Conversely, ppGpp levels after combined treatment with serine hydroxamate and thiostrepton (~87.3 pmol/OD_600 nm_) remained close to baseline (~81.6 pmol/OD_600 nm_) demonstrating an inhibitory effect of thiostrepton on induction of ppGpp biosynthesis by serine hydroxamate. ppGpp levels slightly increased after treatment with thiostrepton alone (~122 pmol/OD_600 nm_), but this increase was not significant (*p* value = 0.0286).

### *Neisseria gonorrhoeae* after treatment with serine hydroxamate: Transcript profile of genes involved in basic cellular functions and cell metabolism

Treatment of gonococci with serine hydroxamate has been shown to be an excellent strategy for inducing ppGpp accumulation, and to analyze the reorganization of the transcriptome during the SR. Therefore, after setting the experimental conditions to induce the SR with this compound, we evaluated the effects of serine hydroxamate treatment on the transcriptional profile of *N. gonorrhoeae* T9 by RNA-seq to identify specific changes in the expression of genes involved in basic cellular function and cell metabolism. In RNA-seq experiments, gonococci were grown in GC broth with Polyvitox up to mid logarithmic phase (0.5 OD_600nm_), and then serine hydroxamate was added to a final concentration of 1 mg/mL. Bacteria were then collected at different time points (10 and 30 min) to extract total RNA, and to evaluate the transcriptional profile compared to the untreated bacteria at the same time points. At 10 min, up-regulated and down-regulated transcripts (fold change ≥2) were, respectively, 158 and 209 (Table 1). At 30 min, the number of up-regulated and down-regulated transcripts increased to 247 and 312, respectively (Table 1). 117 and 179 transcripts were, respectively, up-regulated and down-regulated at both 10 and 30 min (Supplementary Tables S1, S2).

**Table 1:** Overview of RNA-seq data.

	Serine hydroxamate		Thiostrepton		Serine hydroxamate + Thiostrepton	
	10 min	30 min	10 min	30 min	10 min	30 min
Up-regulated (fold change ≥ 2)	158	247	32	159	106	238
Up-regulated (log2 fold change ≥ 2)	14	40	2	18	8	28
Down-regulated (fold change ≥ 2)	209	312	37	170	152	310
Down-regulated (log2 fold change ≥ 2)	15	57	5	15	15	72

Serine hydroxamate and thiostrepton were used at the concentration of 1 mg/mL and 3.75 μg/mL, respectively.

Forty-one and thirty transcripts were, respectively, up-regulated and down-regulated in serine hydroxamate-treated bacteria only at 10 min (Supplementary Table S1), and should be considered related to early response to treatment with serine hydroxamate. Among the 41 transcripts up-regulated at 10 min, it may be noted a conspicuous number of tRNAs, RNase P RNA component (*rnpB*), and a number of transcripts related to sulfur metabolism and response to oxidative stress (*cysE*, *cysT*, *msrAB*), transposable or phage elements, and type II toxin-antitoxin modules (antitoxin FitA).

**Figure 3:**
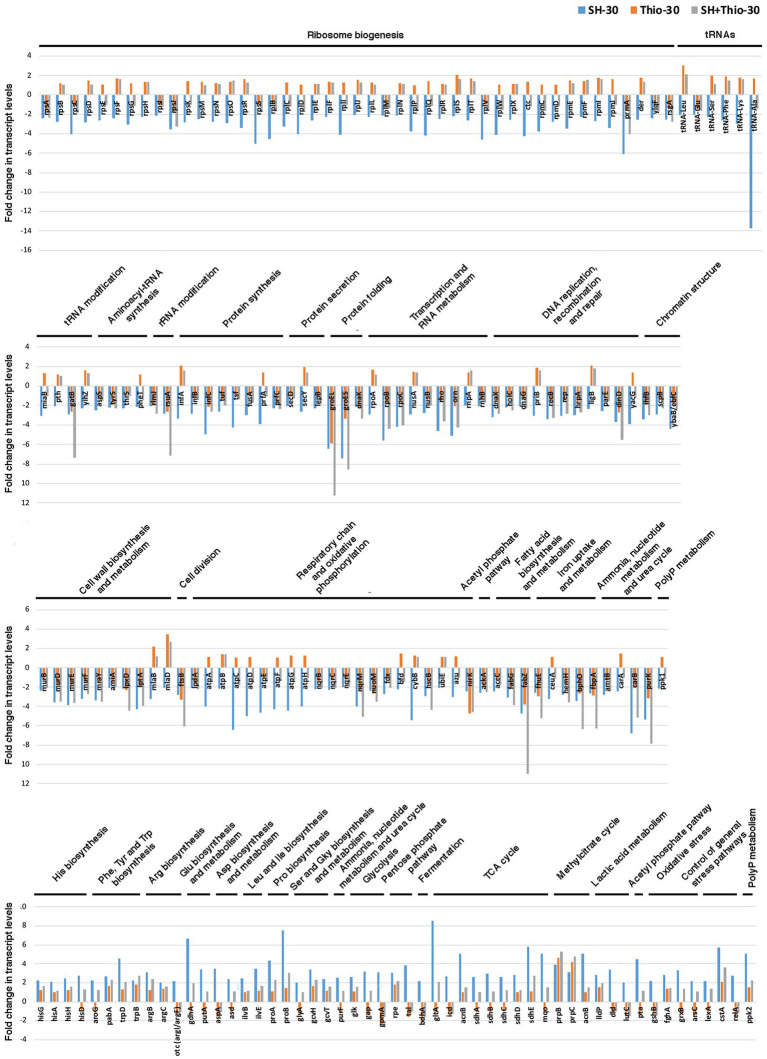
Transcript levels of genes involved in basic cellular function and cell metabolism. Fold changes were calculated by comparing the read values of the treated samples (with serine hydroxamate at the concentration of 1 mg/mL, thiostrepton at the concentration of 3.75 μg/mL, or both drugs for 30 min) with the read values of the control samples. The list only includes genes with fold change ≥2 in transcript levels after treatment with serine hydroxamate for 30 min as compared to control (untreated) sample. Fold changes were calculated with DESeq2.

At 30 min (Figure 3; Supplementary Table S2) the SR appeared to be fully induced with down-regulation of many transcripts involved in: (i) ribosome biogenesis, tRNA and tRNA modification, aminoacyl-tRNA synthesis, protein synthesis and secretion; (ii) protein folding; (iii) Transcription and RNA metabolism; (iv) DNA replication, recombination and repair and chromatin structure; (v) cell wall biosynthesis and metabolism; (vi) cell division; (vii) Respiratory chain and oxidative phosphorylation; (viii) Fatty acid biosynthesis and metabolism; (ix) iron uptake and metabolism. Genes involved in ammonia uptake, ammonia, nucleotide metabolism and urea cycle were also down-regulated.

Conversely, consistent with full induced SR, in bacteria treated with serine hydroxamate we observed at the same time point (30 min) up-regulation of many transcripts involved in: (i) amino acid biosynthesis and metabolism (histidine, arginine, proline, leucine and isoleucine biosynthesis; glutamic acid, aspartic acid, serine and glycine biosynthesis and metabolism), which paralleled down-regulation of transcripts involved in ribosome biogenesis and protein synthesis; (ii) central carbon metabolism (glycolysis, pentose phosphate pathway, fermentation, TCA cycle, methylcitrate cycle, lactic acid metabolism), which parallels the down-regulation of transcripts involved in respiratory chain and oxidative phosphorylation (Figure 3; Supplementary Table S2).

It may be also noted up-regulation of *pta* encoding phosphate acetyltransferase, which parallels the down-regulation of *ackA* encoding acetate kinase, demonstrating an involvement of the SR in regulation of the acetyl phosphate pathway. Furthermore, we observed up-regulation of genes involved in oxidative stress and anti-oxidant defense including: (i) *gshB* coding for glutathione synthase; (ii) *fghA* encoding S-formylglutathione hydrolase; (iii) Genes encoding glutaredoxins (*grxB*, *arsC*) and Dyp-type peroxidase. Genes coding for recombination protein NinB and IS*1595* transposase were also up-regulated.

Among up-regulated genes involved in control of general stress pathways we found: (i) *lexA* coding for LexA family transcriptional regulator; (ii) *cstA* encoding carbon starvation protein A. (iii) *relA* encoding RelA protein. Regarding RelA, similar to other β-proteobacteria, *N. gonorrhoeae* possesses two long RSHs, RelA (WX61_RS01875 in strain 35/02), and SpoT (WX61_RS01485). In bacteria treated with serine hydroxamate we also noticed the up-regulation of *ppk2* encoding polyphosphate kinase 2 (PPK2), in spite of down-regulation of *ppk1* coding for polyphosphate kinase 1 (PPK1). This discrepancy may be due to differences in kinetic properties of PPK1 and PPK2. In fact, although both PPK1 and PPK2 are able to catalyze reversible polyphosphate biosynthesis from ATP, PPK2 catalyzes preferentially polyphosphate-driven nucleotide phosphorylation (Motomura et al., 2014). The up-regulation of *ppk2* could therefore counteract the decrease of the ATP and GTP pool during the SR.

Overall, the observed changes in the transcript profile of many genes involved in basic cellular functions and cell metabolism confirm the efficacy of serine hydroxamate in inducing the SR in *N. gonorrhoeae* under the experimental conditions used in this study.

### Transcript profiling after treatment with thiostrepton

Before evaluating the potential ability of thiostrepton to inhibit the changes in the transcriptional profile associated with the induction of the stringent response with serine hydroxamate, we analyzed the changes induced by treatment of *N. gonorrhoeae* T9 with thiostrepton alone. This experiment would also have given us the possibility to distinguish the changes specifically associated with the stringent response from those generically associated with perturbation of protein synthesis caused by either serine hydroxamate or thiostrepton. To this purpose, gonococci were grown in GC broth with Polyvitox up to mid logarithmic phase (0.5 OD_600nm_), and then thiostrepton was added to a final concentration of 3.75 μg/mL corresponding to the MBC. Bacteria were then collected at different time points (10 and 30 min) to extract total RNA, and to evaluate the transcriptional profile compared to the untreated bacteria at the same time points. At 10 min, up-regulated and down-regulated transcripts (fold change ≥2) were, respectively, 32 and 37 (Table 1). At 30 min, the number of up-regulated and down-regulated transcripts increased to 159 and 170, respectively (Table 1). Only 10 and 9 transcripts were, respectively, up-regulated and down-regulated at both 10 and 30 min (Supplementary Tables S3, S4).

Among the transcripts up-regulated at both 10 and 30 min, it may be noted many tRNAs, and two transcripts coding for ComEA proteins involved in genetic transformation (Supplementary Table S3). Other tRNAs were up-regulated only at 10 min, while tRNA-Pro (RS07420) was up-regulated at 10 min and down-regulated at 30 min, and an additional tRNA-Pro (RS08240) was down-regulated only at 10 min. At 10 min, we also found the up-regulation of the *iscSUA* operon transcript, which encodes proteins belonging to the housekeeping ISC biogenesis pathway that is involved in maintenance and repair of the Fe-S protein pool. This finding may suggest an induction of oxidative stress. Among the transcripts down-regulated at both 10 and 30 min, it may be noted *nirK* (encoding copper-containing nitrite reductase that in pathogenic *Neisseria* supports respiration when oxygen becomes limiting), and *rusA* (coding for RusA family crossover junction endodeoxyribonuclease) transcripts along with transcripts coding for cytochrome-c peroxidase, valine-pyruvate transaminase, phospholipase D family protein, and restriction endonuclease subunit S (Supplementary Table S3). At 10 min, it may be also noted the down-regulation of *ykgO*, which codes for type B 50S ribosomal protein L36 that maps very close to L11 (the target of thiostrepton) on the physical map of large ribosomal subunit.

Effects of thiostrepton on ribosome dynamics at 30 min were more marked, with down-regulation of *infC* encoding translation initiation factor IF-3. In contrast, we noticed up-regulation of genes coding for proteins involved in ribosome biogenesis (*rpmA*, *rpmH*, *rplS*, *rplU*, *rimP*), aminoacyl-tRNA synthesis (*metG*, *serS, gatB*), rRNA and tRNA modification (*rimJ*, *tilS, tsaD, trmD*), protein synthesis (*infA*) and secretion (*ffs, secB*), suggesting an opposite trend with respect to serine hydroxamate treatment (Figure 3, Supplementary Tables S4, S7). The opposite trend between thiostrepton and serine hydroxamate treatments was confirmed by down-regulation, in bacteria treated with thiostrepton, of genes involved in: (i) amino acid biosynthesis (histidine biosynthesis: *hisB*; tryptophan biosynthesis: *trpC, trpF*; arginine biosynthesis: *argA*, *argH*, *argJ*; leucine and isoleucine biosyntesis: *leuB, ilvC*; cysteine biosynthesis: *cysK*); (ii) central carbon metabolism (glycolysis: *gap*, *gpmA, eno;* pentose phosphate pathway: *tkt*; fermentation: *bdhA*; TCA cycle: *sucD, aceF*; Figure 3, Supplementary Tables S4, S7).

In contrast, similar to what was observed in serine hydroxamate-treated bacteria, 30 min after treatment with thiostrepton we found down-regulation of genes coding for proteins involved in: (i) protein folding (*groEL*, *groES, dnaK*); (ii) transcription and RNA metabolism (*rpoD, orn*); (iii) DNA recombination and repair (*uvrB, dinD, rusA*); (iv) cell wall biosynthesis and metabolism (*lptG, lpxA; lpxD*); (v) cell division (*ftsB*); (vi) respiratory chain and oxidative phosphorylation (*nuoL*, *nuoM*, *nqrM*, *ubiM*, *nirK*); fatty acid biosynthesis and metabolism (*fabG*, *fabD, fabZ*; Figure 3, Supplementary Tables S4, S7). In addition, as well as in serine hydroxamate-treated bacteria, we found up-regulation of genes coding for IS*1595* family transposase, and proteins involved in methylcitrate cycle (*prpB*, *prpC*) and lactic acid metabolism (*lldP*).

### Transcript profiling after treatment with both serine hydroxamate and thiostrepton

We then evaluated the effects of combined treatment with serine hydroxamate and thiostrepton on transcriptional profile of *N. gonorrhoeae* T9 compared to untreated bacteria at the same time points. In this experiment, gonococci were grown in GC broth with Polyvitox up to mid logarithmic phase (0.5 OD_600 nm_), and then the two compounds were added to a final concentration of 1 mg/mL and 3.75 μg/mL, respectively. After 10 min of treatment, up-regulated and down-regulated transcripts (fold change ≥2) were, respectively, 106 and 152 (Table 1; Supplementary Table S5). After 30 min, the number of up-regulated and down-regulated transcripts increased to 238 and 310, respectively (Table 1; Supplementary Table S6). At 10 min, 78 out of the 106 up-regulated transcripts (72.9%), and 102 out of the 152 down-regulated transcripts (67.3%) were found to be, respectively, up-regulated and down-regulated also in sample treated with only serine hydroxamate for 10 min, suggesting a large overlap.

However, it may be interesting to note the absence, among down-regulated transcripts in bacteria treated with both serine hydroxamate and thiostrepton, of many genes coding for ribosomal proteins, and translational factors, and the subunits of the F_0_F_1_ ATPase (Supplementary Table S5), which were down-regulated in the bacteria treated with only serine hydroxamate compared to untreated bacteria (Supplementary Table S1). Indeed, down-regulation of these genes is a hallmark of the SR, so this finding confirmed the ability of thiostrepton to counteract the SR induction by serine hydroxamate.

This hypothesis was further supported by RNA-seq data after 30 min of treatment with both drugs (Figure 3; Supplementary Tables S6, S7). At this time point, 103 out of the 238 up-regulated transcripts (43.3%), and 141 out of the 310 down-regulated transcripts (45.5%) were found to be, respectively, up-regulated and down-regulated also in sample treated with only serine hydroxamate for 30 min. Moreover, 106 out of the 238 up-regulated transcripts (44.5%), and 144 out of the 310 down-regulated transcripts (46.4%) were found to be, respectively, up-regulated and down-regulated also in sample treated with only thiostrepton for 30 min.

Noteworthy, 38 out of the 238 up-regulated transcripts (15.9%), and 66 out of the 310 down-regulated transcripts (21.3%) were found to be, respectively, up-regulated and down-regulated in all conditions (serine hydroxamate, thiostrepton, serine hydroxamate + thiostrepton) compared to untreated bacteria. This set of up/down-regulated transcripts may be associated with general (translational) stress, regardless of the antibiotic that generated it. Among up-regulated transcripts we found those coding for proteins involved in competence for natural transformation (*comEA*) and recombination (*ninB*), transposition (IS*1595* family transposase), toxin-antitoxin systems (immunity 41 family proteins), carbon starvation control (*cstA*), lactate utilization (*lldP*), and methylcitrate cycle (*prpB*, *prpC*).

Among down-regulated transcripts in all conditions, it may be noted those coding for chaperonins (*groES*, *groEL*, *dnaK*), respiratory chain (*nqrB*, *nqrC*, *nqrE*, *nqrM*, *nuoM*, *fdk*, *hscB*, *nirK*), fatty acid metabolism (*accC*, *fabG*, *fabZ*), DNA replication, recombination and repair (*dnaX*, *holC*, *dnaG*, *recB*, *rep*, *hrpA*, *parE*, *dinD*), iron uptake and metabolism (*hemK*, *bphO*, *fbpA*), rRNA (16S rRNA, 23S rRNA, 5S rRNA), rRNA and tRNA modification (*gatB*), translation (*infB*, *infC*, *tuf*, *tsf*, *fusA*), and cell division and cell wall biosynthesis (*ftsB*, *murB*, *murD*, *murE*, *murF*, *mraY*, *amiA*, *lpxD*, *lptA*). It can also be noted the down-regulation of genes encoding restriction modification systems, which could further enhance the competence for natural transformation by alleviating the restriction-modification system barrier.

As compared to bacteria treated with only serine hydroxamate for 30 min, in bacteria treated with both serine hydroxamate and thiostrepton it may be noted that genes involved in amino acid biosynthesis were not or were only moderately up-regulated compared to untreated bacteria (Figure 3; Supplementary Tables S6, S7). At the same time, among down-regulated transcripts, we noted the absence of genes coding for ribosomal proteins, translational factors, and the subunits of the F_0_F_1_ ATPase (Figure 3; Supplementary Table S6), which were down-regulated in bacteria treated with serine hydroxamate (Figure 3; Supplementary Table S2). This result confirms once again the ability of thiostrepton to inhibit the induction of the SR.

In contrast, in bacteria treated with both drugs some transcripts involved in ribosome biogenesis were up-regulated, including *rplU* (encoding L21), *rpmA* (encoding L27), *rpmB* (encoding L28), *rpmG* (encoding L33), and *rpmH* (encoding L34; Supplementary Table S6). Up-regulation of these transcripts may be a consequence of thiostrepton treatment, as *rplU*, *rpmA* and *rpmH* were up-regulated also in bacteria treated with only thiostrepton (Supplementary Table S4), and, more interestingly, L28 and L33, L27 and L34 establish direct contacts, respectively, with E-site tRNA and P-site tRNA (Roberts et al., 2008), suggesting that modulation of these transcripts can be directly related to the action of thiostrepton. A consequence of the action of thiostrepton may be also the up-regulation of many tRNAs, *rimP* (coding for ribosome maturation factor RimP), and many genes involved in the biogenesis and assembly of the outer membrane (*mlaE*, *mlaD*, *waaA*, *rfaE2*, *lpxK*, *lap*; Supplementary Table S6).

**Figure 4:**
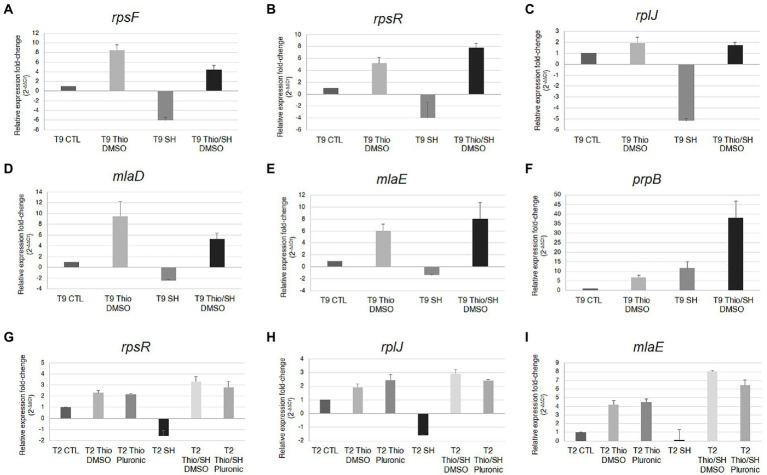
Real-time RT-PCR analysis of *rpsF*, *rpsR*, *rplJ*, *mlaD*, *mlaE*, and *prpB* transcripts. *N. gonorrhoeae* strains T9 **(A–F)** or T2 **(G–I)** strains were grown in GC broth up to middle logarithmic phase, and then treated for 30 min with serine hydroxamate and/or thiostrepton (at the concentration of 1 mg/mL and 3.75 μg/mL, respectively) or no drug. Semiquantitative analysis of *rpsF*, *rpsR*, *rplJ*, *mlaD*, *mlaE*, and *prpB* transcripts normalized to 16S rRNA was carried out by real-time RT-PCR. Values are means of duplicate experiments with standard deviations.

The results of RNA-seq were validated by semiquantitative analysis of *rpsF*, *rpsR*, *rplJ*, *mlaD*, *mlaE*, and *prpB* transcripts normalized to 16S rRNA by real-time reverse transcriptase PCR (real time RT-PCR; Figure 4). These experiments were performed using both the *N. gonorrhoeae* T9 strain (Martin et al., 1986), used for RNA-seq analysis, and another clinical isolate, the T2 strain (Martin et al., 1986), to see if the observed results could be extended to other strains as well. Furthermore, the analysis of the *rpsR*, *rplJ*, and *mlaE* transcripts in the T2 strain after treatment with thiostrepton was carried out by resuspending the compound in the stock solution with either DMSO (used for the treatment of T9 strain) or Pluronic^®^ F-127 (Kudrin et al., 2017), to see if the observed results could be affected by the mode of resuspension and delivery of thiostrepton. The results of real time RT-PCR with the limited set of selected genes were consistent with RNA-seq results. Moreover, very similar results were observed with T9 and T2, and no significant differences were detected by resuspending thiostrepton with DMSO or Pluronic® F-127 (Figure 4).

### Non-metric multidimensional scaling and enrichment analyses

**Figure 5:**
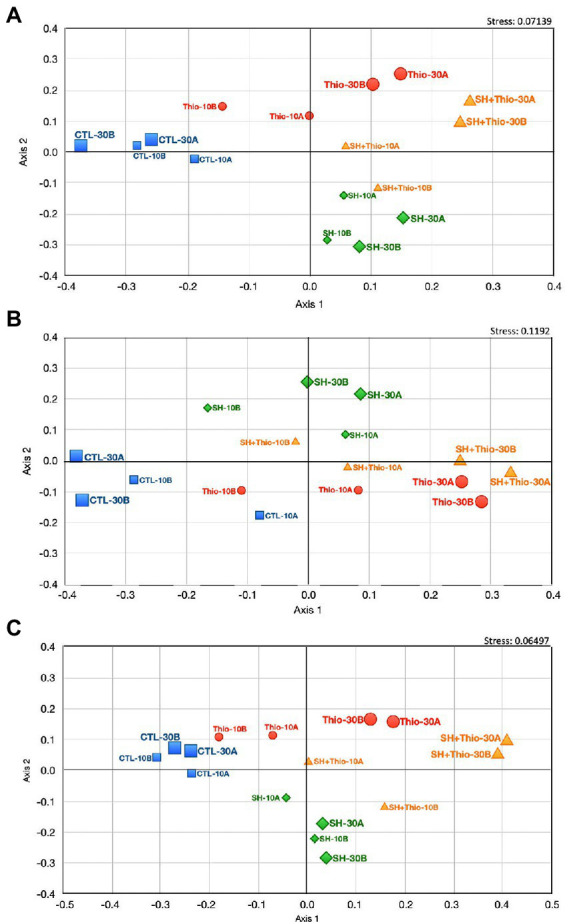
Non-metric multidimensional scaling (NM-MDS) was performed using the Bray–Curtis similarity index and the raw RNA-seq data. Samples treated with 1 mg/mL serine hydroxamate (SH) are shown in green, samples treated with 3.75 μg/mL thiostrepton (Thio) are shown in red, and samples treated with both 1 mg/mL serine hydroxamate and 3.75 μg/mL thiostrepton (SH + Thio) are shown in orange. The effect of these compounds was measured 10 min or 30 min after treatment. CTL are the control samples (untreated bacteria) in blue. **(A)** NM-MDS performed with the entire RNA-seq dataset. **(B)** NM-MDS performed by processing RNA-seq data referring to toxin-antitoxin systems. **(C**) NM-MDS performed by processing RNA-seq data referring to genes involved in host-pathogen interaction.

**Figure 6:**
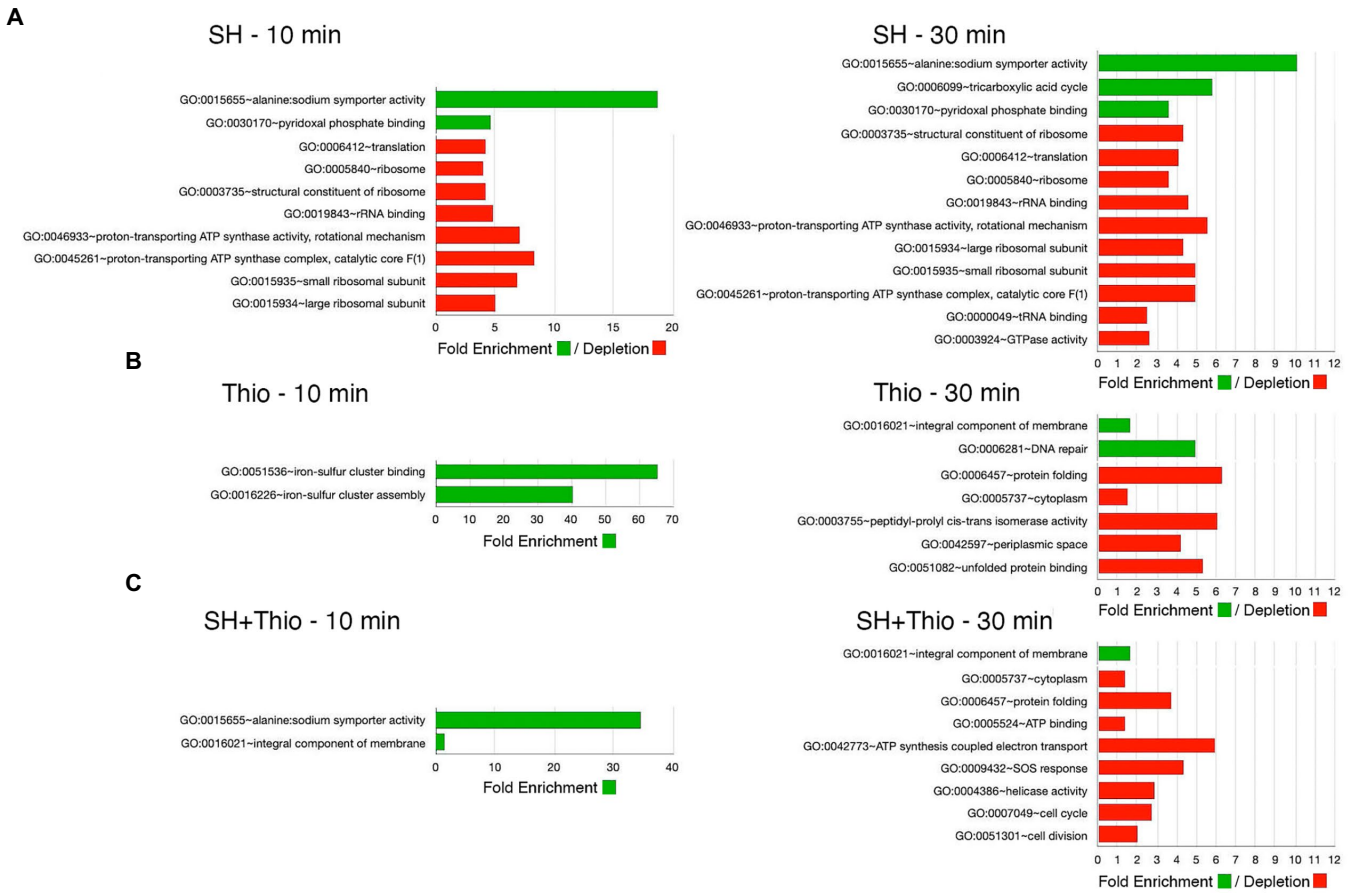
Gene Ontology (GO) term enrichment analysis. Gene enrichment and functional annotation analysis was performed with DAVID. The analysis was performed using the list of up-regulated and down-regulated genes obtained by RNA-seq. **(A)** Samples treated with serine hydroxamate (SH). **(B)** Samples treated with thiostrepton (Thio). **(C)** Samples treated with serine hydroxamate and thiostrepton (SH + Thio). Red: down-regulated GO terms. Green: up-regulated GO terms.

In order to provide a general overview of the (dis-)similarity between the different bacterial samples, we performed a non-metric multidimensional scaling (NM-MDS) analysis, using all transcriptomic data (Figure 5A). This analysis showed a good separation of the samples subject to different treatments, and, above all, highlighted that bacterial samples treated with both serine hydroxamate and thiostrepton for 10 min, and even more for 30 min, were located closer to samples treated with thiostrepton than that treated with serine hydroxamate. This conclusion was supported by Gene Ontology (GO) term enrichment analysis using automated DAVID (Figure 6; Supplementary Table S8) classification tool. Compared to untreated bacteria, after 30 min of serine hydroxamate treatment, GO term enrichment analysis showed statistically significant (*p* < 0.05) enrichment in the following categories: alanine:sodium symporter activity; tricarboxylic acid cycle; pyridoxal phosphate binding. In contrast, the analysis showed depletion in: structural constituent of ribosome; translation; ribosome; rRNA binding; proton-transporting ATP synthase activity, rotational mechanism; large ribosomal subunit; small ribosomal subunit; proton-transporting ATP synthase complex, catalytic core *F* (1); tRNA binding; GTPase activity. Compared to untreated bacteria, in bacteria treated with thiostrepton for 30 min we found statistically significant (*p* < 0.05) enrichment only in the category “integral component of membrane.” In bacteria treated with thiostrepton, the analysis showed depletion in: protein folding; cytoplasm; peptidyl-prolyl cis-trans isomerase activity; periplasmic space; unfolded protein binding. Compared to untreated bacteria, in bacteria treated with both serine hydroxamate and thiostrepton for 30 min we found an enrichment/depletion pattern more similar to that of bacteria treated only with thiostrepton than to that of bacteria treated only with serine hydroxamate at the same time point. In fact, as well as in bacteria treated only with thiostrepton, the only enriched category was “integral component of membrane,” while the depleted categories were: protein folding; cytoplasm; ATP binding, ATP synthesis coupled electron transport; SOS response; helicase activity; cell cycle; cell division. “Protein folding” and “cytoplasm” categories were depleted also in bacteria treated only with thiostrepton. In contrast, in bacteria treated with both drugs, no overlap was found with the enriched or depleted categories in bacteria treated only with serine hydroxamate.

### Expression of toxin-antitoxin modules after treatment with serine hydroxamate and/or thiostrepton

**Figure 7:**
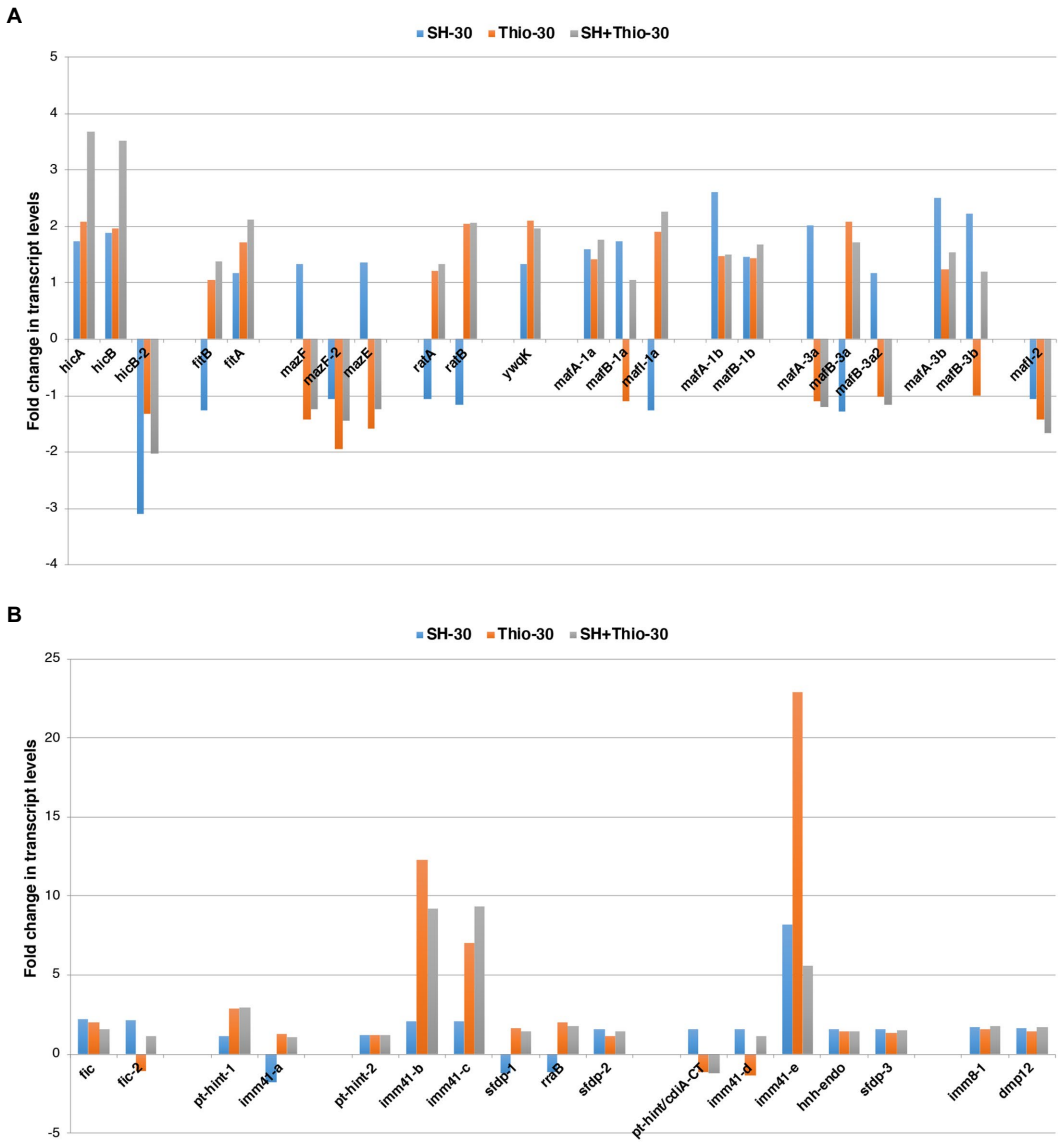
Transcript levels of genes involved in toxin-antitoxin systems. **(A)** Transcript levels of genes coding for known toxin-antitoxin modules. **(B)** Transcript levels of genes coding for immunity proteins or proteins possibly involved in unknown toxin-antitoxin systems. Fold changes were calculated by comparing the read values of the treated samples (with serine hydroxamate at the concentration of 1 mg/mL, thiostrepton at the concentration 3.75 μg/mL, or both drugs for 30 min) with the read values of the control samples. Fold changes were calculated with DESeq2.

There is evidence that stress conditions can induce the expression of toxin-antitoxin systems transcriptionally, although this induction may not result in activation of toxicity (LeRoux et al., 2020). Thus, we went into more detail on RNA-seq data to analyze their expression after 30 min of treatment with serine hydroxamate, thiostrepton, or both drugs (Figure 7).

Preliminarily, we identified the toxin-antitoxin modules in the genome of the reference gonococcal strain 35/02 (Supplementary Table S9). A total of 39 genes forming toxin-antitoxin modules were identified, some of which were presented as multiple paralogs. 22 genes formed toxin-antitoxin modules already characterized in bacteria, including *hicA/hicB* (*hicA, hicB, hicB-2*), *fitB/fitA*, *mazF/mazE* (*mazF, mazF-2, mazE*), *ratA/ratB*, *mafA/mafB/mafI* (*mafA-1a, mafB-1a, mafI-1a, mafA-1b, mafB-1b, mafA-3a, mafB-3a, mafB-3a2, mafA-3b, mafB-3b, mafI-2*), *ywq* (encoding an orphan antitoxin; Figure 7A; Supplementary Table S9). 17 genes encoded immunity proteins or proteins possibly involved in unknown toxin-antitoxin systems (here indicated as *fic*, *fic-2*, *pt-hint-1, pt-hint-2, pt-hint/cdiA-CT, imm41-a, imm41-b, imm41-c, imm41-d, imm41-e, imm8-1, sfdp-1, sfdp-2, sfdp-3, rraB, hnh-endo, dmp12*) (Figure 7B; Supplementary Table S9).

RNA-seq data showed that 19 of the 39 genes (22 of the toxin-antitoxin module genes described above and 17 of the immunity genes described above) were up-regulated after any treatment with the drugs compared to untreated bacteria at the same time point. In contrast, only 3 of the 39 genes (22 genes of the toxin-antitoxin module genes described above and 17 of the immunity genes described above) were down-regulated after any treatment compared to untreated bacteria. The remaining 17 genes exhibited different trends after treatment (Figure 7). Noteworthy, 13 of these 17 genes (*fitB, mazF, mazE, ratA, ratB, mafI1-a, mafA-3a, mafB-3a, mafB-3a2, imm41-a, sfdp-1, rraB, pt-hint/cdiA-CT*) showed opposite trends in bacteria treated with serine hydroxamate and in bacteria treated with thiostrepton compared to untreated bacteria, and the same trends in bacteria treated with thiostrepton and in bacteria treated with both serine hydroxamate and thiostrepton. Moreover, it may be interesting to note that, in contrast, only 3 of the 39 genes (22 of the toxin-antitoxin module genes described above and 17 of the immunity genes described above) were down-regulated after any treatment, compared to untreated bacteria, *mazF, mazE, mafA-3a, mafB-3a2* were up-regulated in gonococci after treatment with serine hydroxamate, and down-regulated in bacteria treated with thiostrepton and in bacteria treated with serine hydroxamate plus thiostrepton. In contrast, *mafI1-a*, which encodes an antitoxin, exhibited the opposite trend. These results may suggest that the expression of *mazF/mazE* and *mafA/mafB/mafI* may be subject to positive transcriptional control by the SR, and may be inhibited by thiostrepton.

NM-MDS analysis showed that, particularly after 30 min of treatment, bacteria treated with both serine hydroxamate and thiostrepton showed a much more similar pattern of toxin-antitoxin gene expression to that of thiostrepton-treated bacteria compared to that of bacteria treated with serine hydroxamate (Figure 5B).

### Expression of genes involved in host-pathogen interaction after treatment with serine hydroxamate and/or thiostrepton

**Figure 8:**
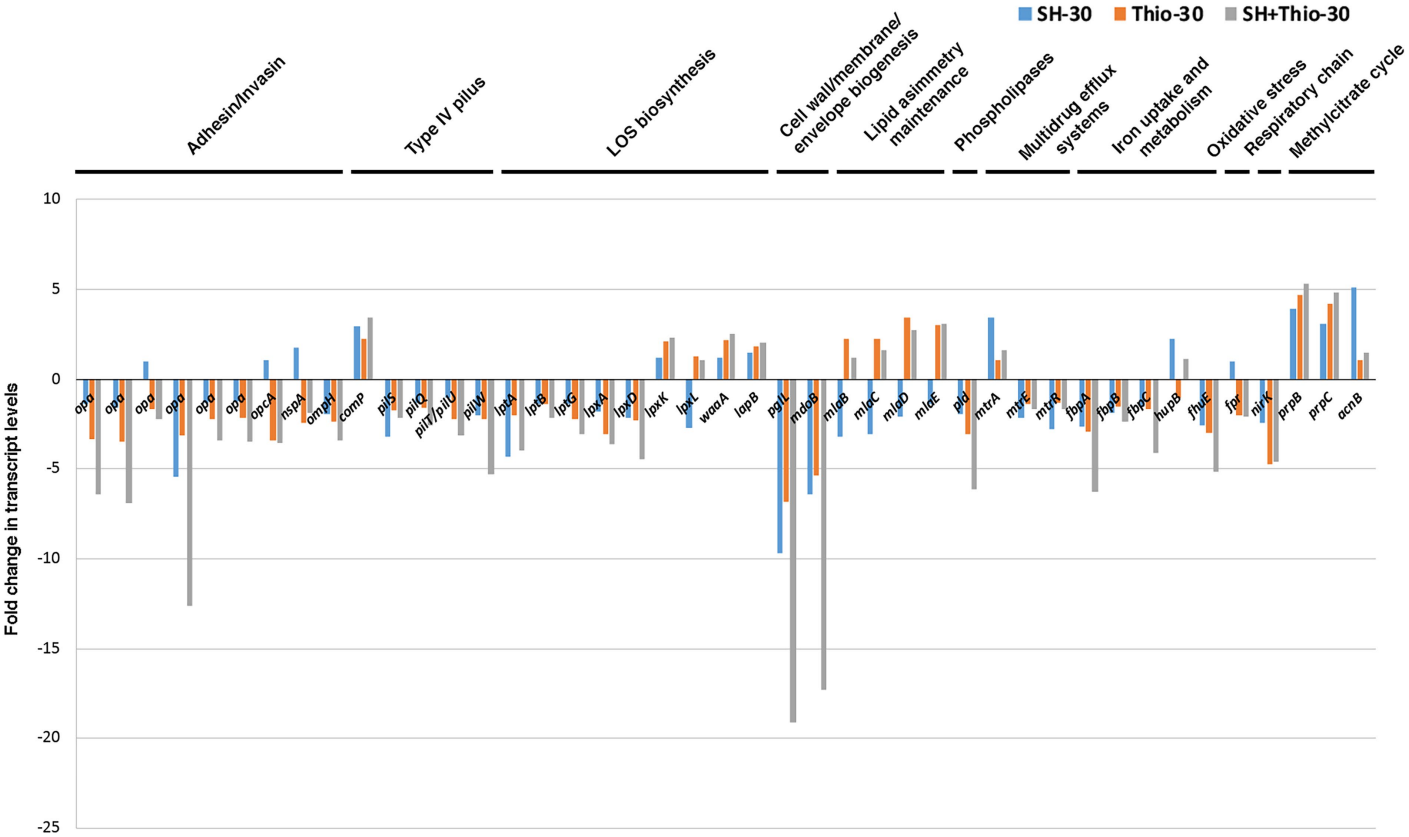
Transcript levels of genes involved in host-pathogen interaction. Fold changes were calculated by comparing the read values of the treated samples (with serine hydroxamate at the concentration of 1 mg/mL, thiostrepton at the concentration of 3.75 μg/mL, or both drugs for 30 min) with the read values of the control samples. Fold changes were calculated with DESeq2.

We went into more detail on RNA-seq data to analyze the expression of genes involved in host-pathogen interaction after 30 min of treatment with the different drugs (Figure 8; Supplementary Table S10). *N. gonorrhoeae*, an obligate human pathogen, encodes a relatively small repertoire of well-established pathogenesis and colonization factors. This microorganism uses an array of surface structures including type IV pili, lipooligosaccharide (LOS), porin, and opacity (Opa) proteins to adhere to host cells, occasionally invade host cells and evade the immune system (Edwards and Butler, 2011; Quillin and Seifert, 2018).

The analysis of RNA-seq data showed that expression of most paralogous genes encoding Opa proteins were not affected by serine hydroxamate treatment (except RS04325), while their expression was significantly down-regulated after treatment with thiostrepton or combined treatment with serine hydroxamate and thiostrepton compared to untreated bacteria (Figure 8; Supplementary Table S10). The combined treatment with the two drugs also resulted in down-regulation of genes coding for pilus secretin PilQ (*pilQ*), pilus assembly protein ATPase PilT/PilU (*pilT/pilU*), pilus biogenesis/stability protein PilW (*pilW*), and silent type IV pili major pilin PilS (*pilS* RS04430) transcript, compared to untreated bacteria. Expression of these genes was differently affected by treatment with either serine hydroxamate (down-regulated: *pilS* RS04430; unchanged: *pilT/pilU*) or thiostrepton (down-regulated: *pilT/pilU*; unchanged: *pilS* RS04430). In contrast, *comP*, encoding Type IV pilus minor pilin protein ComP involved in genetic competence for transformation, was up-regulated after all treatments compared to untreated bacteria.

Compared to untreated bacteria at the same time point, the gene encoding phospholipase D (*pld*) was also down-regulated after the combined treatment with the two drugs. Phospholipase D plays a central role in signaling events and intracellular trafficking during the gonococcal infection of cervical epithelia (Edwards and Butler, 2011). Regarding the genes involved in LOS biosynthesis, many of them (*lptA*, *lptB*, *lptG*, *lpxA*, *lpxD, pglL*) were down-regulated after the combined treatment with the two drugs, while *lpxK*, *waaA* and *lapB* were up-regulated, and *lpxL* was unchanged. *lptA*, *lptG*, *lpxA*, *lpxD, and pglL* expression was also down-regulated after treatment with thiostrepton alone, while that of *lpxK and waaA* was up-regulated, and that of *lptB*, *lpxL* and *lapB* remained substantially unchanged. Expression of most of these genes involved in LOS biosynthesis was significantly affected (>2-fold) after treatment with serine hydroxamate.

During infection, efflux pumps MtrC–MtrD–MtrE and FarA–FarB protect the gonococcus from antimicrobials, fatty acid stress and antimicrobial effectors of the innate defence (Warner et al., 2007). Regarding *mtr* genes, we only found variation in bacteria treated with serine hydroxamate, with up-regulation of *mtrA* (encoding transcriptional activator MtrA), and down-regulation of both *mtrE* (encoding outer membrane subunit MtrE), and *mtrR* (encoding transcriptional repressor MtrR) compared to untreated bacteria. Moreover, in bacteria treated with thiostrepton we observed strong up-regulation of genes encoding proteins involved in Mla pathway (*mlaB*, *mlaC*, *mlaD*, *mlaE*), which maintains outer membrane lipid asymmetry by transporting phospholipids between the inner and outer membranes, and has been shown to aid in antimicrobial resistance (Low and Chng, 2021; Tang et al., 2021). *mlaD* and *mlaE* were also up-regulated in gonococci treated with both thiostrepton and serine hydroxamate, while *mlaB*, *mlaC* and *mlaD* were down-regulated in bacteria treated only with serine hydroxamate. Up-regulation of the Mla pathway following treatment with thiostrepton may be part of a de-toxification response.

NM-MDS analysis confirmed that, after 30 min of treatment, bacteria treated with both serine hydroxamate and thiostrepton had a gene expression pattern of host-pathogen interaction factors much more similar to that of bacteria treated with thiostrepton than that of bacteria treated with serine hydroxamate (Figure 5C).

## Discussion

Local reduction of growth rates has been associated with tolerance against various antibiotics, and there is evidence that persister cells may be formed in a fraction of bacterial population experiencing stressful nutritional conditions that lead to activation of the stringent response with temporary increases in (p)ppGpp levels and consequent reprogramming of gene expression (Fernández et al., 2011; Kaldalu et al., 2020; Pacios et al., 2020). Indeed, down-regulation of genes involved in ribosome biogenesis and protein synthesis, DNA replication, and cell wall biosynthesis during the SR could make treatment with aminoglycoside antibiotics, fluoroquinolones or β-lactams, respectively, less effective.

In this study, we used the serine hydroxamate to induce the stringent response in *N. gonorrhoeae* strain T9. Growth of this strain was completely inhibited at a serine hydroxamate concentration [concentrations are for DL-serine hydroxamate; note that only the L form is active (Tosa and Pizer, 1971a)] of 1 mg/mL (8.32 mM; Figure 2). The sensitivity of the *N. gonorrhoeae* T9 to DL-serine hydroxamate was similar to that of other Gram-negative bacteria including *Salmonella enterica* sv. Typhimurium LT2, whose exponential growth was completely inhibited at 10 mM DL-serine hydroxamate (Shand et al., 1989). Noteworthy, in initial studies in which, however, only the L form of serine hydroxamate was used, a much higher sensitivity was reported in *E. coli* K-12, whose exponential growth was completely inhibited at 0.64 mM L-serine hydroxamate concentration (Tosa and Pizer, 1971b; Pizer and Merlie, 1973). The discrepancy between the sensitivity to serine hydroxamate found in *N. gonorrhoeae* and in *S. enterica* compared to *E. coli* in the initial studies can therefore be attributed to the form (DL or L) of serine hydroxamate used in the experiments.

Concerning the baseline ppGpp level and the extent of ppGpp level increase following serine hydroxamate treatment, comparisons with *E. coli* and other Gram-negative bacteria are more difficult to make. Indeed, in *E. coli* ppGpp basal levels are inversely correlated with growth rates of bacteria on different media (Lazzarini et al., 1971; Ryals et al., 1982; Sarubbi et al., 1988), and this relationship was observed over a wide range of ppGpp levels from about 16 to 80 pmol/OD_600 nm_ and a range of doubling times from about 40 to 200 min (Lazzarini et al., 1971; Ryals et al., 1982). In the T9 strain of *N. gonorrhoeae* growing in GC broth with a doubling time of about 80 min we found a basal ppGpp of ~81.6 pmol/OD_600 nm_ (Figure 2). This finding may suggest a higher baseline ppGpp level in *N. gonorrhoeae* than in *E. coli*, which may reflect a different physiological response to the ppGpp or different ppGpp dynamics in two bacteria. Consistent with this view is evidence that *N. gonorrhoeae relA* null mutants exhibit significantly depressed growth rate even in rich medium, while *E. coli relA* null mutants grow with wild type characteristics (Fisher et al., 2005).

The high basal ppGpp levels in this gonococcus strain could also explain the limited increase (approximately 2.6-fold) in ppGpp levels after treatment with serine hydroxamate (Figure 2). In fact, in *E. coli* the magnitude of the increase in the pGpp level after the induction of the stringent response is inversely correlated with the baseline ppGpp level (Ryals et al., 1982). After severe amino acid starvation, starting from a baseline level of about 80 pmol/OD600 nm, about 400 pmol/OD_600nm_ of ppGpp was reached (an increase of about 5-fold), while starting from a baseline level of about 16 pmol/OD_600nm_ of ppGpp more than 800 pmol/OD600 nm of ppGpp was achieved (over 50-fold increase). The 2.6-fold increase in ppGpp levels measured in the gonococcus after treatment with serine hydroxamate is, therefore, compatible with the results obtained in *E. coli*.

The results of the transcriptome analysis of a clinical isolate of *N. gonorrhoeae* after treatment with serine hydroxamate provided a list of genes that are controlled by the SR. The list includes genes involved in metabolism, cell machine functions, virulence, genome plasticity, and tolerance or persistence to antibiotic treatment. From an ecological point of view, it can be noted that unfavorable nutritional conditions that may induce the SR, including amino acid starvation, are not uncommon in the different microenvironments of gonococcal colonization in the human host, such as gonococcal-containing vacuoles (Edwards and Butler, 2011). Therefore, it is conceivable that persister cells are formed in a fraction of bacterial population during the host-pathogen interaction. Furthermore, there is evidence that exposure to antibiotics can, by itself, trigger the synthesis of (p)ppGpp in various bacteria such as *Staphylococcus aureus* exposed to β-lactams (Kim et al., 2013; Geiger et al., 2014).

Since there is evidence that tolerance and persister cell formation is strongly induced under condition that favors the activation of stress pathways, these pathways represent good targets for new drugs. In this study we analyzed the effects of thiostrepton, a well-known thiopeptide that inhibits the induction of the SR (Cundliffe and Thompson, 1981; Fehr and Richter, 1981; Kudrin et al., 2017, 2018), on growth and transcriptome of *N. gonorrhoeae*. MIC, MBC and kill curves demonstrate that thiostrepton *in vitro* is highly effective against *N. gonorrhoeae*, and does not seem to induce tolerance, persistence or adaptive resistance (Figure 1). This may be due to an inhibitory effect on induction of the SR as suggested by transcriptomic data (Figures 3, 5) and real-time RT-PCR analysis (Figure 4). Furthermore, treatment with thiostrepton determined a down-regulation of many gonococcal genes involved in the host-pathogen interaction and virulence (Figure 8).

Among the pathogenesis and colonization factors, genes encoding Opa proteins and type IV pili were significantly down-regulated after treatment with serine hydroxamate and/or thiostrepton compared to untreated bacteria (Figure 8). Opa proteins are abundant outer membrane proteins that mediate adherence after initial contact by type IV pili, and immune evasion by phase and antigenic variation. Interactions between Opa proteins and carcinoembryonic antigen-related cell adhesion molecule (CEACAM) receptors and other molecules, like heparin sulfate, are considered essential for colonization of the mucosal epithelium of the genital tract and other sites of infection both in women and men (Quillin and Seifert, 2018).

Although the molecular mechanism underlying down-regulation of genes encoding Opa proteins and type IV pili after treatment with the drugs will deserve further investigation, we hypothesize that iron homeostasis and response to oxidative stress may be involved. Indeed, the expression of *opa* genes is iron-repressed (Jackson et al., 2010), and is regulated by ferric uptake regulatory protein (Fur) that binds to the promoter regions of all *opa* genes (Sebastian et al., 2002). In gonococcus, *fur* expression is also iron-repressed (Agarwal et al., 2008; Jackson et al., 2010), and, interestingly, we observed that *fur* (WX61_RS00815) was down-regulated after 30 min treatment with serine hydroxamate compared to untreated bacteria at the same time point (Supplementary Table S2). Moreover, we also found strong down-regulation of *fbpA* after treatment with serine hydroxamate, thiostrepton or both antibiotics, and moderate down-regulation of *fbpB* and *fbcC* after treatment with both serine hydroxamate and thiostrepton (Figure 8). FbpA-FbpB-FbpC transporter shuttles heme and ferric iron from transferrin through the periplasm cytoplasmic membrane (Edwards and Butler, 2011; Quillin and Seifert, 2018), and its expression is regulated by Fur and directly related to the degree of iron restriction (Sebastian et al., 2002; Jackson et al., 2010). Thus, we propose that treatment of the bacteria with serine hydroxamate, thiostrepton, or both drugs, which results in down-regulation of respiratory chain genes (Figure 3), may lead to repression of the Fur regulon to reduce intracellular iron levels to prevent Fenton chemistry and ROS generation. Similar links between iron homeostasis, aerobic respiratory chain activity, ROS generation and the SR have been shown in other bacteria (Vinella et al., 2005; Kim et al., 2018; Fritsch et al., 2020). In particular, in *Vibrio cholerae* (p)ppGpp accumulation led to repression of FbpA (Kim et al., 2018). Up-regulation of genes involved in oxidative stress and anti-oxidant defense including *gshB*, *fghA*, *grxB*, and *arsC* in gonococci treated for 30 min with serine hydroxamate compared to untreated bacteria (Figure 3) may be indicative of an induction of oxidative stress during the SR.

In the context of metabolic adaptation of the gonococcus to the exposure to the drugs, it should be underlined the strong up-regulation, after treatment with serine hydroxamate and/or thiostrepton, of genes involved in the methylcitrate pathway (*prpB*, *prpC*) that in *Mycobacterium tuberculosis* is thought to be involved in many processes including optimal lactate and pyruvate metabolism, propionate and butyrate metabolism, survival during oxidative stress, acid stress and starvation, and persistence (Serafini et al., 2019). In pathogenic Neisseriae the *prp* gene cluster, which encodes the enzymes for the methylcitrate cycle, a proprionate kinase and a putative propionate transporter, is located within a genomic island that is absent in commensal Neisseriae such as *Neisseria lactamica* (Catenazzi et al., 2014). The presence of this cluster enables *N. meningitidis* to utilize propionic acid as a supplementary carbon source during growth, particularly under nutrient poor growth conditions, and also to avoid toxicity of propionate in the adult oral cavity, which is rich in propionic acid-generating bacteria (Catenazzi et al., 2014).

Treatment with serine hydroxamate and/or thiostrepton also resulted in transcriptional changes in toxin-antitoxin modules (Figure 7). Indeed, there is evidence that stressful conditions may induce transcriptional expression of toxin-antitoxin systems, although, as noted above, increased transcription does not necessarily imply that the toxin is produced and activated (LeRoux et al., 2020). Therefore, the concept that stress can induce the expression of toxin-antitoxin systems and, consequently, the persistent phenotype appears unsupported based on the evidence in the literature. Furthermore, it is increasingly evident the involvement of most of these systems in processes other than tolerance or persistence toward antibiotics, including defense against bacteriophages (Guegler and Laub, 2021), and bacterial virulence (Lobato-Márquez et al., 2016; Kamruzzaman et al., 2021).

In this context, it may be interesting to note the expression pattern of *mafA/mafB/mafI* system, which appears to be transcriptionally up-regulated by serine hydroxamate, and down-regulated by thiostrepton. In particular, MafB are strain-specific toxins with EndoU ribonuclease activity, which are secreted by *Neisseria* spp. and are involved in interbacterial competition (Arenas et al., 2015; Jamet et al., 2015). *mafB* genes are usually located downstream of *mafA* encoding MafA proteins that, interestingly, have a role in virulence with reported activities in adhesion and transcytosis of pathogenic, but are also involved in secretion of MafB, while genes immediately downstream of *mafB* encode a specific immunity protein (MafI) forming a toxin-antitoxin module (Arenas et al., 2020).

More specifically, MafA is a relatively less studied adhesin that in *Neisseria meningitidis* mediates the interaction with human brain microvascular endothelial cells, and triggers the activation of TLR-dependent pathways and cytokine response. In addition, it is able to induce genes involved in cell surface modifications, endocytosis, extracellular matrix remodulation and anoikis/apoptosis (Káňová et al., 2019). Thus, it would be interesting to verify whether increased transcriptional levels of *mafA* after treatment with serine hydroxamate leads to an increase of MafA protein levels. This would support the hypothesis that the SR could induce a series of host cell responses relevant to gonococcal pathogenesis.

## Materials and methods

### Bacterial strains and growth conditions

In this study, we used two urogenital clinical isolates of *N. gonorrhoeae* from female patients, named, respectively, T9 (a serotype 9 strain), and T2 (a serotype 2 strain; Martin et al., 1986). These strains were grown, from frozen stocks (−80°C), on GC agar base (OXOID) supplemented with 1% (v/v) Polyvitox (OXOID) at 37°C in a 5% CO_2_ incubator. The liquid medium used for growth was GC broth whose composition (per liter) is as follows: 15 g proteose peptone, 0.5 g corn starch, 4 g K_2_HPO_4_, 1 g KH_2_PO_4_, 5 g NaCl, 1% (v/v) Polyvitox (OXOID).

Thiostrepton (MIC 0.9 μg/mL; MBC 3.75 μg/mL), ampicillin (MIC 0.6 μg/mL; MBC 1.25 μg/mL), gentamicin (MIC 3.75 μg/mL; MBC 7.5 μg/mL), nalidixic acid (MIC 1.875 μg/mL; MBC 3.35 μg/mL), rifampicin (MIC 0.23 μg/mL; MBC 0.9 μg/mL), tetracycline (MIC 0.05 μg/mL; MBC 0.8 μg/mL), and DL-serine hydroxamate (MIC 1 mg/mL; MBC 4 mg/mL; all provided by Sigma-Aldrich) were used at the required concentration (values in brackets) in GC broth. Liquid cultures in GC broth were incubated at 37°C on rotary shaker at 200 rpm.

### Minimum inhibitory concentration, minimum bactericidal concentration, and kill curve experiments

The MICs of thiostrepton, ampicillin, gentamicin, nalidixic acid, rifampicin, tetracycline, and serine hydroxamate against *N. gonorrhoeae* T9 were determined by the macrodilution method, according to the instructions of the Clinical and Laboratory Standards Institute (CLSI) (Clinical and Laboratory Standards Institute, 2018). The procedure was performed in 13-mL plastic tubes with 1 mL GC broth and repeated three times for each antibiotic on independent assays. Positive controls (with bacteria and no drug) and negative controls (without bacteria and drug) were run simultaneously. After incubation for 24 h at 37°C, OD_600nm_ value in each treated well was determined using a spectrophotometer (Onda V-10 Plus). The MIC was defined as the minimum concentration of antibiotic leading to no significant increase of OD_600nm_ value. Stock solutions of drugs were prepared prior to each experiment by dissolving the powder in distilled water for ampicillin (50 mg/mL), gentamicin (10 mg/mL), and serine hydroxamate (50 mg/mL), in ethanol 50% solution for rifampicin (5 mg/mL), and tetracycline (5 mg/mL), in 0.15 M NaOH for nalidixic acid (25 mg/mL), and in dimethyl sulphoxide for thiostrepton (10 mg/mL). Stock solution of thiostrepton was prepared in DMSO for all experiments. In addition, stock solution of thiostrepton was also prepared in 0.1% Pluronic® F-127 (Sigma-Aldrich) as reported by Kudrin et al. (2017), and used when required, as specified. The desired final concentrations were obtained by serial two-fold dilutions of GC broth, starting with the highest concentration. Since the stock solutions of some antibiotics were prepared with ethanol, NaOH or dimethyl sulfoxide, it was previously verified that the use of these substances at the final concentrations in GC had no effect on gonococcal growth.

The MBC of each antibiotic was defined as the minimum concentration of antibiotic leading to no viable cell in the tube. To evaluate the cell viability, the total volume of each inoculated macrodilution tube was spread on GC agar plate, after being washed with phosphate-buffered saline (PBS) twice, and the MBC value was determined after 24 h of incubation at 37°C.

For time-kill curve experiments, single colonies of *N. gonorrhoeae* T9, cultured on GC agar plate overnight, were resuspended into GC liquid medium at OD_600nm_ of 0.05. The suspensions were incubated at 37°C with shaking for 3 h to reach OD_600nm_ of 0.5, corresponding to exponential growth phase. Next, each antibiotic was separately added to the bacterial cultures at a final concentration corresponding to their MBC values. A control tube containing the same bacterial suspension without antibiotics was also run simultaneously. The tubes were incubated at 37°C for 24 h and 10 μL aliquots were withdrawn at different time intervals (0, 1, 2, 3, 4, 5, 6, and 24 h) after the start of the experiment, and washed with PBS twice, for viable counts. Each 10 μL aliquot was then 10-fold serially diluted in PBS and 10 μL from each dilution were spotted on GC agar plates. The numbers of colony forming units (CFU) were counted after incubation at 37°C in 5% CO_2_ overnight, using the most diluted droplet in which colonies were well separated and easy to count. To overcome the limit of detection of 100 CFU/mL, the total bacterial suspension was plated, when required. Each time-kill experiment was carried out in biological triplicate.

### Adaptive resistance experiments

The adaptive resistance of *N. gonorrhoeae* T2 and T9 was investigated using a gradient approach, as previously described with some modifications (Jahn et al., 2017). Gonococci were inoculated in GC broth tubes containing increasing concentrations (50% or 100% gradient) of thiostrepton and ampicillin and incubated at 37°C with shaking for 24 h. The first tube showing growth below the MIC value was used to inoculate a new set of tubes containing 100% or 50% increasing concentrations of the same antibiotic. This procedure was repeated sequentially for 14 days for each strain, for each antibiotic, and for each gradient. At each passage, the MIC value of thiostrepton or ampicillin was determined.

### ppGpp assay

For intracellular assessment of ppGpp levels, *N. gonorrhoeae* T9 was grown at 37°C with shaking to logarithmic phase (OD_600nm_ = 0.5) in GC liquid medium. Next, 50 mL aliquots of the bacterial suspension were split out into new sterile flasks where no drug (control), serine hydroxamate, both serine hydroxamate and thiostrepton, were added at the final concentration of 1 mg/mL and 3.75 μg/mL (MBC), respectively. The cultures were incubated at 37°C with shaking for 30 min. Then, the suspensions were immediately collected by centrifugation for 0.5 min at 14,000 rpm at 4°C and cells were crushed in 3 mL ice-cooled 2 M formic acid for 30 min, shaking every 10 min. The samples were centrifuged at 10,000 rpm for 10 min at 4°C, and the supernatants were filtered through nitrocellulose (Millipore, 0.25 μm pore size). Filtrated samples were lyophilized and resuspended in 100 μL distilled water.

The intracellular concentration of ppGpp was determined as previously described (Peano et al., 2014) by anion exchange chromatography using an Agilent 1,100 HPLC equipped with Phenomenex Partisil SAX column (10 μm, 250 × 4.60 mm) and a PDA detector. A linear gradient of 100/0 to 0/100 (A/B) was run over 20 min, then buffer B was run for 15 min at a flow rate of 1.2 mL/min. Buffer A consisted of 50 mM K_2_HPO_4_, pH 5.4 and buffer B was 0.5 M KH_2_PO_4_, pH 5.4. Nucleotides were detected at 254 nm. The concentration of ppGpp was expressed relative to bacterial O.D. at 600 nm. More than 95% pure ppGpp standard was obtained from Sigma-Aldrich.

### RNA-seq and bioinformatics analysis

*Neisseria gonorrhoeae* T9 was grown at 37°C with shaking to logarithmic phase (OD_600nm_ = 0.5) in GC liquid medium. Next, aliquots of this bacterial suspension were placed into new tubes where serine hydroxamate (1 mg/mL) and/or thiostrepton (3.75 μg/mL) or no drug (control) were added, and the cultures were incubated at 37°C with shaking for 10 min and for 30 min. For each experimental condition and for each time, total bacterial RNAs were then extracted using RNeasy minikit (Qiagen), according to manufacturer’s instructions. Before extraction, samples were treated with 2 volumes of RNA Protect Bacteria reagent (Qiagen). DNA contamination was avoided by on-column treatment with an RNase-free DNase set (Qiagen) according to manufacturer’s instructions. This procedure was performed in duplicate for each experimental condition and for each time point.

Next, generation sequencing experiments were performed by Genomix4life S.R.L. (Baronissi, Salerno, Italy). RNA concentration in each sample was assayed with a NanoDrop ONE (Thermo Scientific) and its quality assessed with the TapeStation 4,200 (Agilent Technologies). Indexed libraries were prepared from 500 ng of each purified RNA with Ribo-Zero Plus rRNA Depletion kit (Illumina) according to manufacturer’s instructions. Libraries were quantified using the TapeStation 4,200 (Agilent Technologies) and Qubit fluorometer (Invitrogen Co.), then pooled such that each index-tagged sample was present in equimolar amounts, with final concentration of the pooled samples of 2 nM. The pooled samples were subject to cluster generation and sequencing using an Illumina NextSeq 550Dx System (Illumina) in a 2 × 75 paired-end format.

The raw sequence files generated (.fastq files) underwent quality control analysis using FastQC^1^ and the quality checked reads were trimmed with cutadapt (Martin, 2011) and then aligned to the genome of the reference strain 35/02 *Neisseria gonorrhoeae* (GCF_001047275.1) obtained from NCBI using STAR (Dobin et al., 2013) with standard parameters. Differentially expressed mRNAs were identified using DESeq2 (Love et al., 2014). Using the reads mapped to the genome, we calculated the number of reads mapping to each transcript with HTSeq-count.^2^ These raw read counts were then used as input to DESeq2 for calculation of normalized signal for each transcript in the samples, and differential expression was reported as Fold Change.

### Real-time reverse transcriptase PCR

*Neisseria gonorrhoeae* strains T9 or T2 were grown at 37°C with shaking to logarithmic phase (OD_600nm_ = 0.5) in GC liquid medium. Next, aliquots of this bacterial suspension were placed into new tubes where serine hydroxamate (1 mg/mL) and/or thiostrepton (3.75 μg/mL) or no drug (control) were added and the cultures were incubated at 37°C with shaking for 10 min and for 30 min. Total RNAs extracted from bacteria treated for 30 min with serine hydroxamate and/or thiostrepton or no drug were quantified using Nanodrop™ (Thermo Scientific), and 1 μg of each RNA was reverse transcribed by using random primers (2.5 μM) with SuperScript™ III Reverse Transcriptase (Invitrogen). Real-Time PCR was performed on a CFX96 System (Bio-rad) with SsoAdvanced™ Universal SYBR® Green Supermix (Bio-Rad) and the primers listed in Table 2. The real-time PCR protocol used was: 120 s at 95°C for 1 cycle, and 30 s at 94°C, 30 s at 55°C, 30 s at 72°C for 35 cycles. Real-time PCR reactions were run in duplicate and data were normalized to the RNA levels of 16S rRNA, and relative mRNA expression was calculated using the ∆∆Ct method.

**Table 2:** Primers used for real-time PCR.

Name	Sequence
16Suniv-1	5′-CAGCAGCCGCGGTAATAC-3′
16S-r	5′-CTACGCATTTCACTGCTACACG-3′
prpB-f	5’-CAGTGACACGCCGTTTTCAG-3’
prpB-r	5’-CCGGTGCGGACATGATTTTC-3’
mlaD-f	5’-GCGATACGGAAAACCTTGC-3’
mlaD-r	5’-TTTTCTCGGCGAAGCTGG-3’
mlaE-f	5’-ATATGGTCGCGGCTTCTCTG-3’
mlaE-r	5’-GCCATCACGTTCATCGCTTC-3’
rpsF-f	5’-ACCGTTTGCCTCGGTAATC-3’
rpsF-r	5’-TCCTGATCAAAGCGAGCAAG-3’
rpsR-f	5’-AGGCAGGAGTGCCAAGAAG-3’
rpsR-r	5’-CCGCATCACAGGAACGAAGG-3’
rplJ-f	5’-GTTGGTCCGTTGGTTTACGC-3’
rplJ-r	5’-CAGCAACCTGAGCAGCATTC-3’

### Non-metric multidimensional scaling

PAST software (V 4.07) (Hammer et al., 2001) was used to perform multivariate analyses. We performed the NM-MDS according to the Bray–Curtis similarity index using raw RNA-seq data with individual read values for each replicate (Figures 5A,B) transformed as log10. This analysis was also used to analyze (dis-)similarity between the different bacterial samples also in the expression of subsets of genes involved in host-pathogen interaction and toxin-antitoxin systems.

### Go term enrichment analysis

Enrichment analysis was performed using the DAVID webpage^3^ (Sherman et al., 2022). The lists of up-regulated and down-regulated genes were selected by analyzing the fold change reported in the RNA-seq data. The identifier used in the lists was “LOCUS_TAG.” The genomic background used for enrichment calculation and functional annotation was that of *N. gonorrhoeae* strain TUM19854 (NZ_AP023069.1). The analysis was performed using Functional Annotation Tool of DAVID, and the results were analyzed as Functional Annotation Chart. The results were considered significant when the *p* value was <0.05.

## Data availability statement

The datasets presented in this study can be found in online repositories. The names of the repository/repositories and accession number(s) can be found in the article/Supplementary material.

## Author contributions

AT, MC, SR, and AP performed the material preparation, data collection, and investigation. AT, GB, and PA performed the data analysis. PA performed the project administration, supervision, and wrote the first draft of the manuscript. All authors contributed to the study conception and design, commented on previous versions of the manuscript, read and approved the final manuscript.

## Funding

This study was partially supported by grants from the Italian MIUR to PA (PRIN 2017, grant 2017SFBFER; PRIN 2020, grant 202089LLEH) and to CIB (Consorzio Interuniversitario Biotecnologie, DM 587, 08/08/2018; CIB N. 86/19).

## Conflict of interest

The authors declare that the research was conducted in the absence of any commercial or financial relationships that could be construed as a potential conflict of interest.

## Publisher’s note

All claims expressed in this article are solely those of the authors and do not necessarily represent those of their affiliated organizations, or those of the publisher, the editors and the reviewers. Any product that may be evaluated in this article, or claim that may be made by its manufacturer, is not guaranteed or endorsed by the publisher.

## Supplementary material

The Supplementary material for this article can be found online at: https://www.frontiersin.org/articles/10.3389/fmicb.2023.1104454/full#supplementary-material

## Footnotes

^1^ http://www.bioinformatics.babraham.ac.uk/projects/fastqc

^2^ http://www-huber.embl.de/HTSeq

^3^ https://david.ncifcrf.gov/
