# Supplementary material for: Thiostrepton, a resurging drug inhibiting the stringent response to counteract antibiotic-resistance and expression of virulence determinants in Neisseria gonorrhoeae
Source: Front Microbiol. 2023 Feb 23;14:1104454. doi: 10.3389/fmicb.2023.1104454 (PMC9998046; doi:10.3389/fmicb.2023.1104454)
Supplement: Supplementary file 1 [file Data_Sheet_1.docx]

Supplementary Material

**Data Availability Statement**

The original RNA-seq row counts and normalized counts are available in a publicly accessible repository at the following Digital Object Identifier [10.6084/m9.figshare.21976670](https://doi.org/10.6084/m9.figshare.21976670).

# Legends to Supplementary Figures and Tables

**Figure S1.** Adaptive resistance experiments with *N. gonorrhoeae* T2. *N. gonorrhoeae* T2 was inoculated sequentially into 14 sets of tubes containing GC broth with increasing concentrations (50% or 100% as indicated) of thiostrepton. Bacteria growing at the highest antibiotic concentration in each set of tubes were used to inoculate the following set with the same antibiotic for a total of 14 passages. After each passage, the MIC of thiostrepton was determined.

**Table S1.** Comparison of gene expression levels between serine hydroxamate-treated bacteria for 10 min (SH10) and untreated bacteria (CN10). In yellow are indicated the transcripts that were up-regulated in serine hydroxamate-treated bacteria at 10 min, in orange are indicated the transcripts down-regulated in serine hydroxamate-treated bacteria at 10 min. The locus name refers to the annotated genome of the reference *N. gonorrhoeae* strain 35/02.

**Table S2.** Comparison of gene expression levels between serine hydroxamate-treated bacteria for 30 min (SH30) and untreated bacteria (CN30). In yellow are indicated the transcripts that were up-regulated in serine hydroxamate-treated bacteria at 30 min, in orange are indicated the transcripts down-regulated in serine hydroxamate-treated bacteria at 30 min. The locus name refers to the annotated genome of the reference *N. gonorrhoeae* strain 35/02.

**Table S3.** Comparison of gene expression levels between thiostrepton-treated bacteria for 10 min (THIO10) and untreated bacteria (CN10). In yellow are indicated the transcripts that were up-regulated in thiostrepton-treated bacteria at 10 min, in orange are indicated the transcripts down-regulated in thiostrepton-treated bacteria at 10 min. The locus name refers to the annotated genome of the reference *N. gonorrhoeae* strain 35/02.

**Table S4.** Comparison of gene expression levels between thiostrepton-treated bacteria for 30 min (THIO30) and untreated bacteria (CN30). In yellow are indicated the transcripts that were up-regulated in thiostrepton-treated bacteria at 30 min, in orange are indicated the transcripts down-regulated in thiostrepton-treated bacteria at 30 min. The locus name refers to the annotated genome of the reference *N. gonorrhoeae* strain 35/02.

**Table S5.** Comparison of gene expression levels between thiostrepton/serine hydroxamate-treated bacteria for 10 min (Thio+SH10) and untreated bacteria (CN10). In yellow are indicated the transcripts that were up-regulated in thiostrepton/serine hydroxamate-treated bacteria at 10 min, in orange are indicated the transcripts down-regulated in thiostrepton/serine hydroxamate -treated bacteria at 10 min. The locus name refers to the annotated genome of the reference *N. gonorrhoeae* strain 35/02.

**Table S6.** Comparison of gene expression levels between thiostrepton/serine hydroxamate-treated bacteria for 30 min (Thio+SH30) and untreated bacteria (CN30). In yellow are indicated the transcripts that were up-regulated in thiostrepton/serine hydroxamate-treated bacteria at 30 min, in orange are indicated the transcripts down-regulated in thiostrepton/serine hydroxamate-treated bacteria at 30 min. The locus name refers to the annotated genome of the reference *N. gonorrhoeae* strain 35/02.

**Table S7.** Comparison of expression level of gene involved in basic cellular functions and cell metabolism. The list only includes genes with fold change ≥2 in transcript levels after treatment with serine hydroxamate for 30 min as compared to control (untreated) sample. The locus name refers to the annotated genome of the reference *N. gonorrhoeae* strain 35/02.

**Table S8.** GO enrichment analysis. Enrichment analysis was performed using the DAVID webpage (<https://david.ncifcrf.gov/>) as described in Materials and methods. The lists of up-regulated and down-regulated genes were selected by analyzing the fold change reported in the RNA-seq data. The genomic background used for enrichment calculation and functional annotation was that of *N. gonorrhoeae* strain TUM19854 (NZ_AP023069.1).

**Table S9.** Comparison of expression level of gene encoding toxin-antitoxin modules and immunity proteins or proteins possibly involved in unknown toxin-antitoxin systems. The locus name refers to the annotated genome of the reference *N. gonorrhoeae* strain 35/02.

**Table S10.** Comparison of expression level of gene involved in host-pathogen interaction. The locus name refers to the annotated genome of the reference *N. gonorrhoeae* strain 35/02.
