# Supplementary figures and images for: Thiostrepton, a resurging drug inhibiting the stringent response to counteract antibiotic-resistance and expression of virulence determinants in Neisseria gonorrhoeae
Source: Front Microbiol. 2023 Feb 23;14:1104454. doi: 10.3389/fmicb.2023.1104454 (PMC9998046; doi:10.3389/fmicb.2023.1104454)

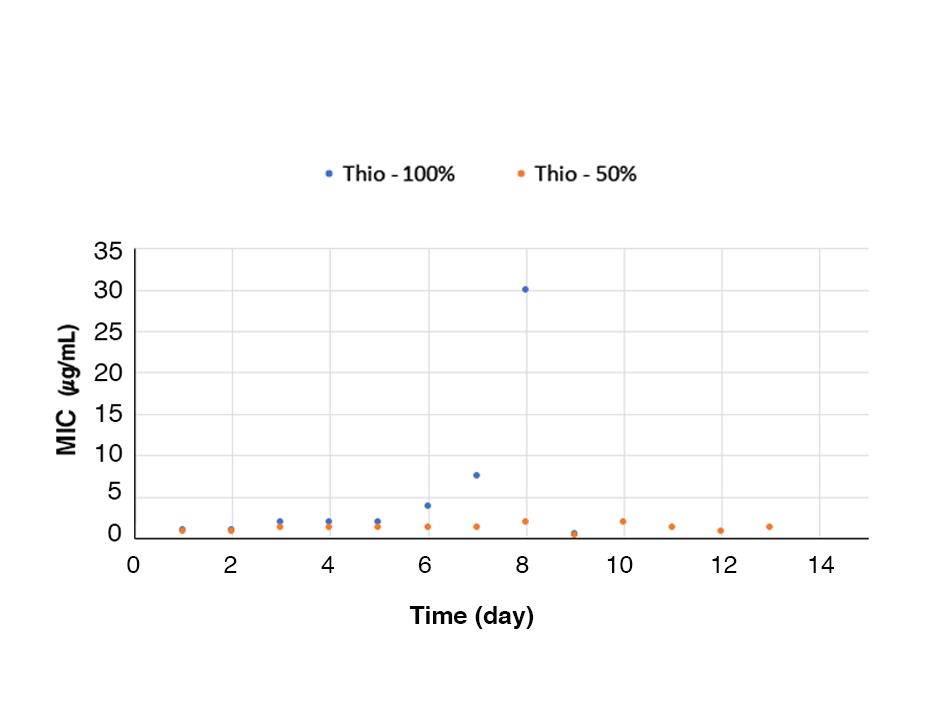

Supplement: Supplementary file 2 [file Image_1.TIF]
